# Bio‐Inspired Structural Color Irises as Physical Unclonable Functions for Anti‐Counterfeiting

**DOI:** 10.1002/advs.202504771

**Published:** 2025-07-10

**Authors:** Kongyu Ge, Yiming Hu, Jiangnan Yan, Jianing Ding, Yifan Gao, Hongyu Yi, Zhan Li, Lingfei Cui, Shaowei Hu, Hongjun Ji, Mingyu Li, Ruobing Bai, Rongpei Shi, Huanhuan Feng

**Affiliations:** ^1^ Sauvage Laboratory for Smart Materials Shenzhen Key Laboratory of Flexible Printed Electronics Technology Harbin Institute of Technology (Shenzhen) Shenzhen 518000 China; ^2^ Harbin Institute of Technology Shenzhen 518000 China; ^3^ State Key Laboratory of Advanced Welding and Joining (Shenzhen) Harbin Institute of Technology Shenzhen 518000 China; ^4^ School of Mechanical Engineering Shanghai Jiao Tong University Shanghai 200240 China

**Keywords:** Anti‐Counterfeiting, Colloidal Photonic Crystals, Iris, Physical Unclonable Functions, Self‐assembly

## Abstract

The global proliferation of high‐value commodities has the intensified demand for advanced anti‐counterfeiting solutions. Physical Unclonable Functions (PUFs) present a viable authentication mechanism, yet existing implementations exhibit three critical limitations: suboptimal authentication accuracy, insufficient multi‐level security architecture, and dependence on costly and specialized equipment for verification. To address these issues, this work utilizes colloidal photonic crystals to fabricate PUF anti‐counterfeiting patterns spanning from the macro‐to‐micro scale. These patterns are integrated with the iris recognition algorithm to construct a multi‐layered optical anti‐counterfeiting system based on the structural color iris. This work experimentally and computationally demonstrates that the “Ring”‐type colloidal photonic crystals originate from the “coffee ring” effect. By modulating the “coffee ring” effect, the size of the colloidal photonic crystal iris patterns can be controlled, enabling switching between “Ring”‐type and “Dot”‐type colloidal photonic crystals. Furthermore, multiple characteristic parameters confirm that the PUF codes derived from encoded “Ring”‐type colloidal photonic crystal images exhibit uniqueness and readability. Finally, a multi‐layered anti‐counterfeiting system is constructed, comprising three components: structural color patterns, embedded “Morse codes” formed by “Ring”‐type and “Dot”‐type colloidal photonic crystals, and optical PUF labels based on the “Ring”‐type colloidal photonic crystals. This cost‐effective system enables low‐threshold, high‐performance structural color anti‐counterfeiting for premium products.

## Graphical abstract

This work employs colloidal photonic crystals to create macro‐to‐micro scale PUF anti‐counterfeiting patterns. Integrated with iris recognition algorithms, it establishes a multi‐layered optical anti‐counterfeiting system based on structural color irises. This cost‐effective approach facilitates low‐threshold, high‐performance anti‐counterfeiting for premium products.

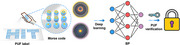

## 1 Introduction

With the continuous development of the economy, various high‐value products are increasingly facing significant challenges in anti‐counterfeiting. Counterfeit products have caused substantial economic losses and posed serious threats to public safety and brand integrity.^[^ ^1^, ^2^, ^3^, ^4^, ^5^, ^6^, ^7^ ^]^ In response, researchers have been dedicated to developing new anti‐counterfeiting technologies, such as metasurface encryption,^[^ ^8^, ^9^, ^10^, ^11^, ^12^ ^]^ holographic color printing,^[^ ^13^, ^14^, ^15^ ^]^ and fluorescent printing.^[^ ^16^, ^17^, ^18^ ^]^ However, these technologies rely on deterministic manufacturing processes, making them potentially vulnerable to replication.^[^ ^19^, ^20^, ^21^, ^22^, ^23^, ^24^, ^25^, ^26^ ^]^ Therefore, there is an urgent need to explore anti‐counterfeiting solutions incorporating randomness that are inherently difficult to forge. Physical Unclonable Functions (PUFs) offer a promising alternative, leveraging random physical characteristics to generate unique and irreproducible identifiers.^[^ ^27^, ^28^, ^29^, ^30^, ^31^, ^32^, ^33^, ^34^, ^35^, ^36^, ^37^ ^]^

Currently, researchers have proposed various PUF anti‐counterfeiting schemes, which can be categorized into Fluorescence PUF,^[^ ^38^, ^39^, ^40^, ^41^, ^42^ ^]^ Raman PUF,^[^ ^43^, ^44^, ^45^, ^46^, ^47^ ^]^ Plasmonic PUF,^[^ ^48^, ^49^, ^50^, ^51^, ^52^, ^53^ ^]^ etc., based on their generation principles. The generation of Fluorescence PUF relies on the emission of fluorescence as a response signal.^[^ ^54^, ^55^, ^56^ ^]^ Common fluorescent materials include rare‐earth‐based phosphors, fluorescent proteins, quantum dots, etc. However, the phenomenon of peak broadening in fluorescence emission leads to signal overlap, significantly reducing the accuracy of identifying response signals. Additionally, fluorescence materials are susceptible to photobleaching, which reduces the practicality of Fluorescence PUF. Raman PUF avoids the peak broadening issue of Fluorescence PUF by utilizing the narrow spectral features of Raman signals to prevent signal overlap.^[^ ^57^, ^58^, ^59^ ^]^ However, the authentication process for Raman PUF requires point‐by‐point scanning and complex spectral decoding equipment, rendering the process slow and the equipment expensive. Plasmonic PUF, while providing narrow emission peaks, also accelerates the identification and authentication process.^[^ ^60^ ^]^ However, its commonly used surface plasmon resonance is highly sensitive to the structural features of nanoparticles. As a result, in Plasmonic PUF authentication systems, even slight changes can lead to different responses, posing a challenge to readout stability. In addition to the above issues, current PUF anti‐counterfeiting schemes lack a structural hierarchy in their design.

**Figure 1:**
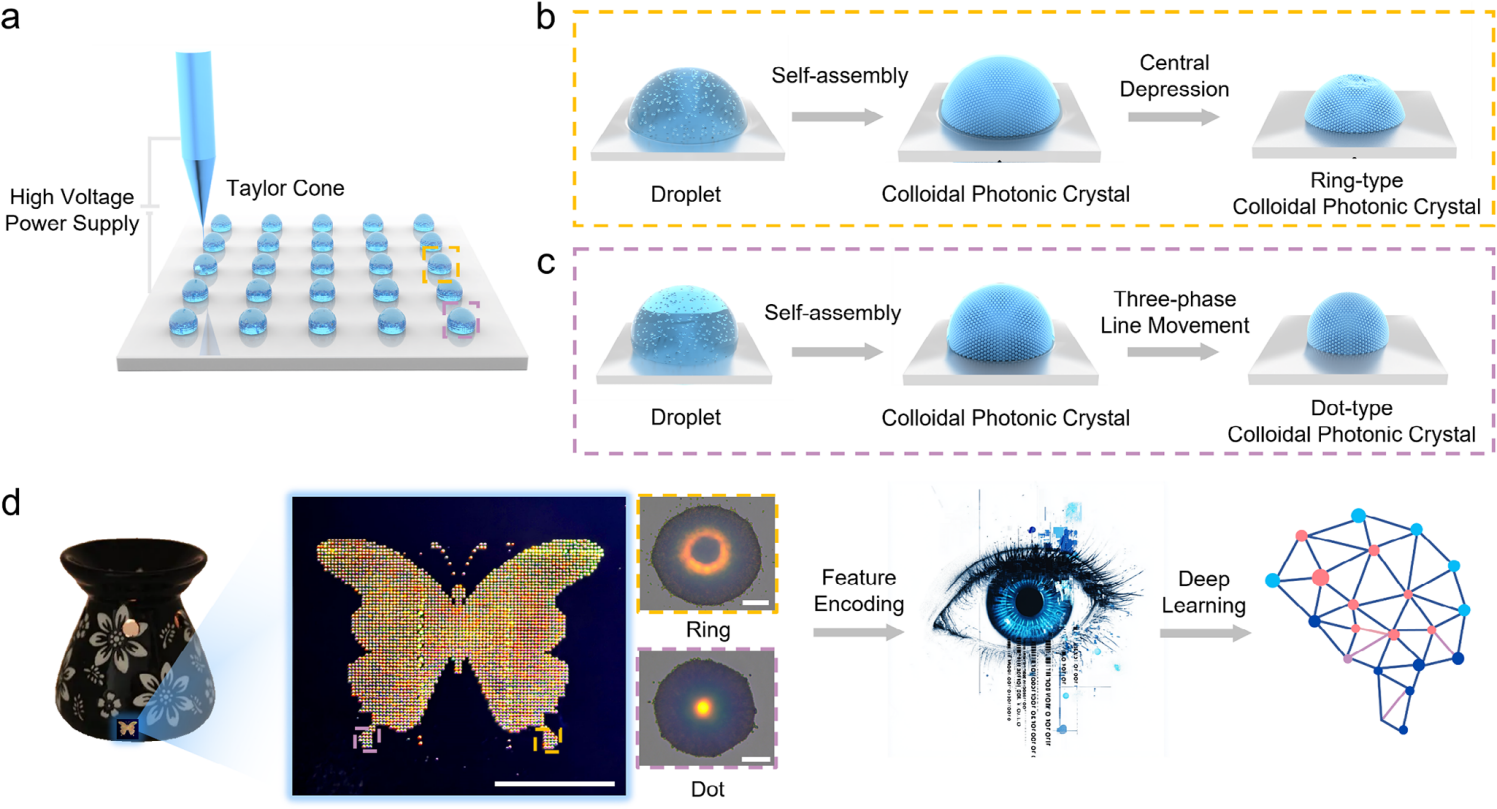
Formation Mechanism of Structural Color iris and Construction of Physically Unclonable Anti‐Counterfeiting System a) Schematic diagram of electrohydrodynamic (EHD) printing; b) Schematic diagram of the formation process of “Ring”‐type colloidal photonic crystals; c) Schematic diagram of the formation process of “Dot”‐type colloidal photonic crystals; d) Schematic diagram of the physically unclonable anti‐counterfeiting system construction (Scale bars: 1 cm for the butterfly pattern, 20 µm for both “Ring”‐type and “Dot”‐type colloidal photonic crystals).

To address the issues in current PUF anti‐counterfeiting solutions, this work leverages the PUF characteristics of the self‐assembly process of colloidal photonic crystals to introduce PUF (“Ring”‐type colloidal crystal), thereby fabricating multi‐layered optical anti‐counterfeiting patterns spanning from the macroscopic to the microscopic scale. Inspired by human iris recognition, the multi‐layer optical anti‐counterfeiting patterns are integrated with iris recognition algorithms to construct a multi‐layer optical anti‐counterfeiting system based on the structural color iris (**Figure** **1**). Initially, the pixel points of the anti‐counterfeiting patterns are constructed through electrohydrodynamic printing and droplet evaporation self‐assembly (Figure 1a). Both experimental and simulation results demonstrate that the ring structure in “Ring”‐type colloidal photonic crystals arises from the reduction of the contact angle after the surface tension of droplet is reconfigured, leading to stable pinning of the three‐phase contact line. This weakens the self‐assembly force of the colloidal nanospheres, making it more difficult for them to accumulate toward the center, thereby forming an ordered ring‐type structure around the periphery. By modulating the strength of the “coffee ring” effect, the size of the biomimetic iris can be controlled, enabling the transition between “Ring”‐type and “Dot”‐type colloidal photonic crystals (Figure 1b,c). Furthermore, multiple characteristic parameters, including bit uniformity, information entropy, similarity, results of NIST tests, Intra‐Hamming Distance (Intra‐HD), Inter‐Hamming Distance (Inter‐HD), False Accept Rate (FAR), and False Rejection Rate (FRR), demonstrate that the PUF codes derived from “Ring”‐type colloidal photonic crystal images exhibit uniqueness and discriminability. Finally, the anti‐counterfeiting system integrates three key components: i) Macroscale structural color patterns, ii) Embedded cryptographic “Morse code” units formed through controlled “Ring”‐type and “Dot”‐type colloidal photonic crystal assemblies and iii) Microscopic optical PUF labels derived from “Ring”‐type colloidal crystal. These three components collectively establish a multi‐layered anti‐counterfeiting system with low recognition thresholds (progressive layers from macroscopic to microscopic) and excellent anti‐counterfeiting performance (0 False Alarm Rate, FAR) (Figure 1d). This system provides an economical and effective solution for combating counterfeit products, further advancing the practical application of structural colors in the anti‐counterfeiting of high‐value products.

## 2 Results and Discussion

### 2.1 Construction Mechanism of Structural Color Iris

Based on previous research,^[^ ^61^ ^]^ colloidal polystyrene (PS‐COOH) nanospheres with a diameter of 265 nm and carboxyl group modification are prepared to construct high‐quality colloidal photonic crystals. These nanospheres are characterized using scanning electron microscopy (SEM) and Fourier‐transform infrared spectroscopy (FTIR). The synthesized PS‐COOH nanospheres exhibit uniform size and high dispersion (monodisperse coefficient of variation less than 0.05), fully meeting the physical size requirements for forming crystalline structural colors (Figure S1, Supporting Information). Furthermore, FTIR spectra reveal characteristic vibrational peaks of carbonyl and hydroxyl groups at 1708 and 3450 cm⁻¹, respectively, confirming the successful modification of carboxyl groups on the surface of the colloidal nanospheres (Figure S2, Supporting Information). Subsequently, the PS‐COOH colloidal nanospheres are formulated into colloidal photonic crystal ink, which is then adapted to an electrohydrodynamic printing device (Figure S3, Supporting Information) and printed onto a hydrophobic silicon wafer substrate (Figure S4, Supporting Information).

**Figure 2:**
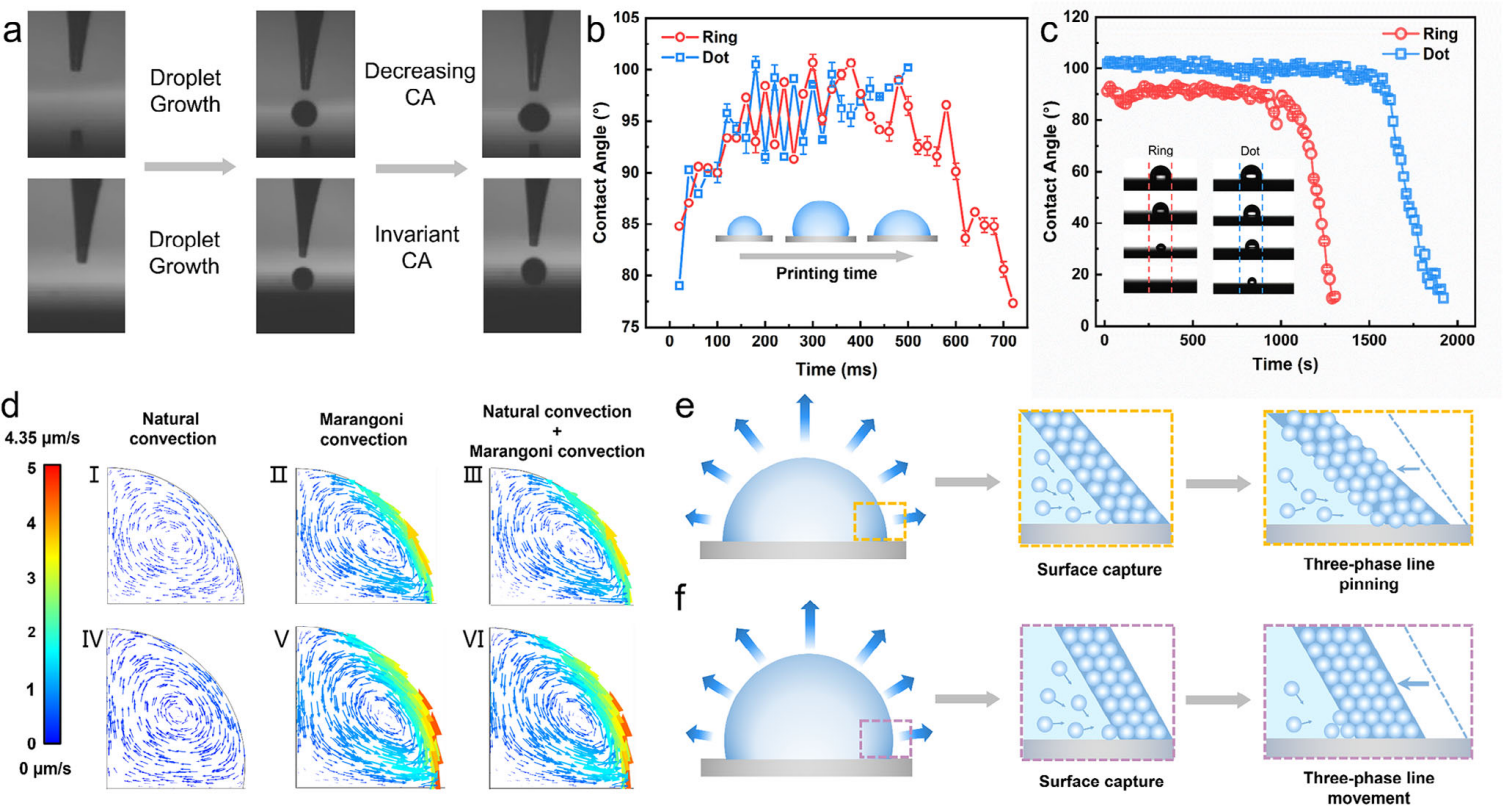
Construction Process and Mechanism of Structural Color Micro‐Domes in “Ring”‐Type and “Dot”‐Type Colloidal Photonic Crystals a) Variation diagrams of “Ring”‐type and “Dot”‐type droplets during EHD printing; b) Measurement of contact angle changes for “Ring”‐type and “Dot”‐type droplets during EHD printing, with insets illustrating droplet shape evolution over printing time; c) Measurement of contact angle changes during the evaporation and self‐assembly process of “Ring”‐type and “Dot”‐type droplets, with insets showing real‐time images of contact angle changes for “Ring”‐type and “Dot”‐type droplets during evaporation and self‐assembly; d) Velocity field simulation results for “Ring”‐type and “Dot”‐type droplets: d‐I) Simulation of "natural convection field" velocity for “Ring”‐type droplets, d‐II) Simulation of "Marangoni flow" velocity for “Ring”‐type droplets, d‐III) Simulation of combined "natural convection + Marangoni flow" field velocity for “Ring”‐type droplets, d‐IV) Simulation of "natural convection field" velocity for “Dot”‐type droplets, d‐V) Simulation of "Marangoni flow" velocity for “Dot”‐type droplets, d‐VI) Simulation of combined "natural convection + Marangoni flow" field velocity for “Dot”‐type droplets; e) Schematic diagram of the formation process of “Ring”‐type colloidal photonic crystals; f) Schematic diagram of the formation process of “Dot”‐type colloidal photonic crystals.

To investigate the formation process of “Ring”‐type and “Dot”‐type colloidal photonic crystals, high‐speed cameras are employed to record the electrohydrodynamic printing process, and the changes in the contact angle of the droplets during this process are observed and measured. As shown in **Figure** **2**a,b, three distinct stages are identified for “Ring”‐type droplets: droplet generation, droplet growth, and droplet surface reconfiguration. During this process, the contact angle of the “Ring”‐type droplets initially increases rapidly from 78° to 100°, then fluctuates and stabilizes between 95° and 100°, before suddenly decreasing from 100° to 77° under the influence of the electric field force (Figure S5 and Movie 1, Supporting Information). For “Dot”‐type droplets, only the first two stages, droplet generation and droplet growth, are observed. The contact angle of “Dot”‐type droplets increases from 78° to 100° and remains stable thereafter(Figure S6 and Movie 2, Supporting Information). This comparison indicates that the key difference between “Ring”‐type and “Dot”‐type droplets during printing lies in the presence or absence of droplet surface reconfiguration. The “Ring”‐type droplets undergo surface reconfiguration, leading to a reduction in the contact angle. Therefore, it can be inferred that the formation of “Ring”‐type colloidal photonic crystals is attributed to the disruption of the original droplet surface tension balance caused by the strong electric field force (depending on the applied potential or printing time) in the later stages of electrohydrodynamic printing. To establish a new equilibrium state for the droplet surface, the surface reconfiguring, causing the three‐phase contact line moves outward, reducing the contact angle and increasing the distance between the liquid surface and the tip, thereby achieving a new equilibrium.

To investigate the influence of this new equilibrium state on the evaporation‐driven self‐assembly of colloidal nanospheres, the evaporation self‐assembly process is observed, and the changes in the droplet contact angle are measured. As shown in Figure 2c, the contact angle of “Ring”‐type droplets (90°) is ≈10° smaller than that of “Dot”‐type droplets (100°), and the pinning time of the three‐phase contact line for “Ring”‐type droplets (1000 s) is ≈50% shorter than that of “Dot”‐type droplets (1500 s). This indicates that the “Ring”‐type droplets in the new equilibrium state exhibit a smaller contact angle and a shorter movement time for the three‐phase contact line. Additionally, it is observed that the complete evaporation time for “Ring”‐type droplets (1250 s) is ≈44% shorter than that for “Dot”‐type droplets (1800 s). This suggests that the colloidal nanospheres in “Ring”‐type droplets have a shorter self‐assembly time.

To further investigate the flow field changes during the evaporation of “Ring”‐type and “Dot”‐type droplets, finite element simulation is conducted, with the modeling process illustrated in Figure S7 (Supporting Information) (details provided in Text S1, Supporting Information). Three models are constructed: “natural convection,” “Marangoni flow,” and “natural convection + Marangoni flow.” Temperature simulations reveal that the temperature profiles of both “Ring”‐type and “Dot”‐type droplets exhibit curvature, but the former is more gradual. This suggests that the flow field velocity in “Ring”‐type droplets is slower compared to that in “Dot”‐type droplets (Figure S8, Supporting Information). Velocity field simulations indicate that the “natural convection” in “Ring”‐type droplets exhibits a clockwise direction with an average velocity of 0.49 µm s^−1^(Figure 2d‐I), which is 33% slower than that in “Dot”‐type droplets (0.65 µm s^−1^) (Figure 2d‐IV). The “Marangoni flow” in “Ring”‐type droplets shows a counterclockwise direction with an average velocity of 0.93 µm s^−1^ (Figure 2d‐II), which is 20% slower than that in “Dot”‐type droplets (1.12 µm s^−1^) (Figure 2d‐V). Consequently, when “natural convection” and “Marangoni flow” are coupled, the combined flow in “Ring”‐type droplets exhibits a counterclockwise direction with an average velocity of 0.78 µm s^−1^ (Figure 2d‐III), which is 24% slower than that in “Dot”‐type droplets (0.97 µm s^−1^) (Figure 2d‐VI). The simulation analysis demonstrates that the internal flow field velocity in “Ring”‐type droplets is lower than in “Dot”‐type droplets Based on the Stokes equation, it can be inferred that the self‐assembly force of colloidal nanospheres in “Ring”‐type droplets is ≈24% weaker than that in “Dot”‐type droplets. This indicates that colloidal nanospheres are less likely to move inward and instead tend to form ring‐like accumulations at the periphery, resulting in a structure similar to the “coffee ring” effect.^[^ ^62^, ^63^ ^]^

Based on the observations of droplet formation and evaporation behavior as well as the corresponding flow field simulations, it can be inferred that the reconfiguration of droplet surface tension balance under electrohydrodynamic printing is the direct cause of the formation of “Ring”‐type colloidal photonic crystals. As illustrated in Figure 2e, after the droplet surface tension is reconfigured, the contact angle decreases, and the three‐phase contact line becomes pinned. This weakens the self‐assembly force of the colloidal nanospheres, making it more difficult for them to accumulate toward the center. Consequently, an ordered ring‐like accumulation forms around the periphery, ultimately resulting in “Ring”‐type colloidal photonic crystals. In contrast, for “Dot”‐type colloidal photonic crystals, as shown in Figure 2f, the larger contact angle and longer movement time of the three‐phase contact line allow the colloidal nanospheres within the droplet to exhibit a stronger self‐assembly force. This leads to tightly ordered inward accumulation, forming an ordered semi‐micro dome structure, and thus creating a central dot‐like pattern.

### 2.2 Regulation of Structural Color Iris

**Figure 3:**
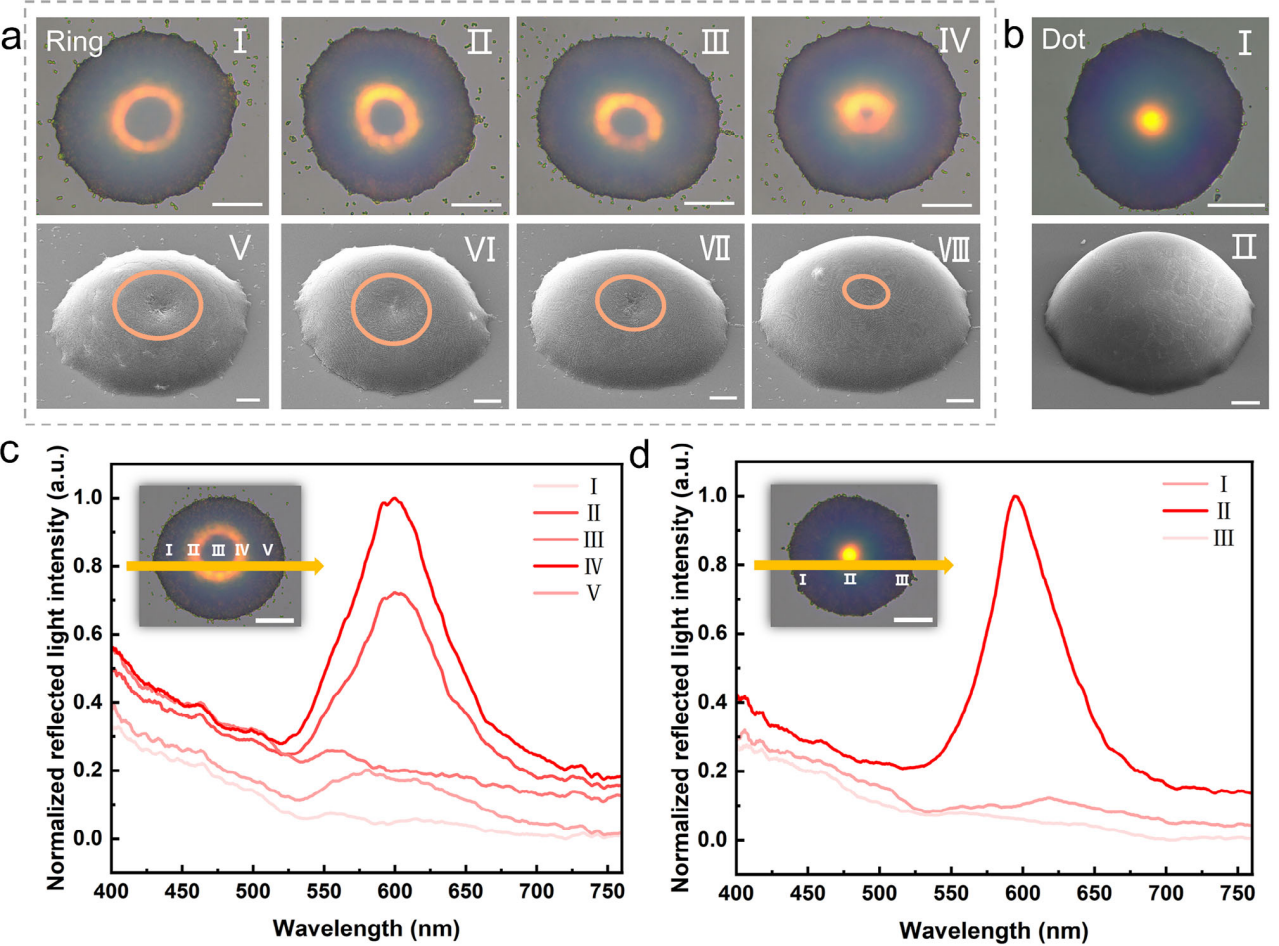
Modulation of “Ring”‐Type and “Dot”‐Type Colloidal Photonic Crystals a) Optical microscopy and SEM images of “Ring”‐type colloidal photonic crystals (Scale bars: 20 µm for optical microscopy, 10 µm for SEM), (a‐I‐a‐IV) optical microscopy images of “Ring”‐type colloidal photonic crystals, (a‐V‐a‐VIII) SEM images of “Ring”‐type colloidal photonic crystals (45° tilted stage); b) Optical microscopy and SEM images of “Dot”‐type colloidal photonic crystals (Scale bars: 20 µm for optical microscopy, 10 µm for SEM), b‐I) optical microscopy image of “Dot”‐type colloidal photonic crystals, b‐II) SEM image of “Dot”‐type colloidal photonic crystals (45° tilted stage); c) Micro‐region reflectance spectra of “Ring”‐type colloidal photonic crystal micro‐domes (Inset: optical microscopy image of “Dot”‐type colloidal photonic crystals, scale bar: 20 µm); d) Micro‐region reflectance spectra of “Dot”‐type colloidal photonic crystal micro‐domes (Inset: optical microscopy image of “Ring”‐type colloidal photonic crystals, scale bar: 20 µm).

Induced by electrohydrodynamic jet printing, the reconfiguration of droplet surface tension equilibrium leads to the formation of two distinct types of colloidal photonic crystals—the “Ring”‐type and the “Dot”‐type, whose microstructures are illustrated in **Figure** **3**a,b, respectively. The “Ring”‐type colloidal photonic crystal exhibits a ring‐like pattern under optical microscopy (Figure 3a‐I), with its corresponding micro‐nano structure displaying a ring‐shaped configuration characterized by higher edges and a lower center (Figure 3a‐V; Figure S9a, Supporting Information). Conversely, the “Dot”‐type colloidal photonic crystal manifests as a dot‐like pattern under optical microscopy (Figure 3‐b‐I), with its corresponding microstructure featuring a central protrusion (Figure 3b‐II) and Figure S9b, Supporting Information). This demonstrates that the different arrangements of colloidal nanospheres directly influence the patterns of colloidal photonic crystal units observed under optical microscopy, which is consistent with the construction mechanism previously proposed.

To further substantiate the aforementioned observations, “Ring”‐type colloidal photonic crystals with varying protrusion positions are constructed. As illustrated in Figure 3a‐I,a‐IV, the widening of the corresponding ring‐like optical pattern is observed as the ring‐like stacking structure gradually shifts from the center outward (indicated by the orange circles in the figure). Additionally, optical microscopy images of the “Ring”‐type colloidal photonic crystals at different AP (aperture angle) values are recorded. As shown in Figure S10 (Supporting Information), as AP values increase, the “Ring” structure exhibits a gradual divergence, while its central line remains unchanged. This indirectly demonstrates that the generation of the “Ring” structural color pattern originates from the ring‐like stacking of colloidal nanospheres, which is consistent with the observations from SEM results.

Furthermore, to delve deeper into the origins of the structural color patterns in colloidal photonic crystals, FIB (focused ion beam) cutting is performed on the colloidal photonic crystals, and the arrangement of colloidal nanospheres on the cross‐section is examined. As shown in Figure S11 (Supporting Information), it is observed that the arrangement of colloidal nanospheres at the “Ring”‐type protrusion is primarily divided into two parts: the upper part, where colloidal nanospheres are neatly arranged into an ordered part, and the lower part, where colloidal nanospheres are neatly ordered. Within the ordered part, two types of arrangements are observed: one is the face‐centered cubic (FCC) arrangement (highlighted in red in Figure S11c, Supporting Information), and the other is the simple cubic (SC) arrangement (highlighted in green in Figure S11c, Supporting Information). The emergence of the SC arrangement may result from the release of minor internal stress. It is these close‐packed structures that form the corresponding structural color patterns of the colloidal photonic crystals (simulation results are shown in Figure S12, Supporting Information).

In summary, the “Ring”‐type and “Dot”‐type colloidal photonic crystals primarily arise from the ordered arrangement of colloidal nanoparticles. The ordered arrays of colloidal nanoparticles form the corresponding “photonic band gaps”, which in turn give rise to the structural color unit patterns (“Ring”‐type or “Dot”‐type). The distinction between the “Ring”‐type and “Dot”‐type colloidal photonic crystals stems from the reconfiguration of surface tension in the droplets. This reconstruction phenomenon is driven by the “coffee ring” effect, resulting in a ring‐like ordered structure.

To quantitatively characterize the differences between the ring‐like structure in “Ring”‐type colloidal photonic crystals and the “Dot”‐type colloidal photonic crystals, micro‐region reflection spectroscopy is conducted. As shown in Figure 3c, in the reflection spectra of the “Ring”‐type colloidal photonic crystals, five micro‐regions are sequentially selected from left to right. The intensity of the reflection spectra exhibits a trend of initially increasing, then decreasing, increasing again, and finally decreasing, which aligns with the ring‐like structure. Additionally, the brightness at the two ends of the same ring is not identical, likely due to the randomness in the stacking of colloidal nanospheres. This randomness provides additional encoding capacity for subsequent PUF label applications. For the “Dot”‐type colloidal photonic crystals, three micro‐regions are sequentially selected from left to right. The intensity of the reflection spectra shows an initial increase followed by a decrease, consistent with the dot‐like structure. These results indicate significant differences in the micro‐region reflection spectral characteristics between the “Ring”‐type and “Dot”‐type colloidal photonic crystals, which will facilitate the construction of anti‐counterfeiting systems based on their corresponding ring‐like and dot‐like patterns.

In addition to regulating the switching between “Ring”‐type and “Dot”‐type colloidal photonic crystals, the strength of the “coffee ring” effect in droplets can also be modulated to construct “Ring”‐type colloidal photonic crystals with varying diameters. As shown in Figure S13 (Supporting Information), it is observed that colloidal photonic crystals with diameters ranging from 39 to 84 µm all form ring‐like structures. However, when the diameter is less than 44 µm, the resulting ring‐like structures are smaller, which may lead to unclear recognition and misjudgment in the construction of PUF labels. Meanwhile, when the diameter exceeds 71 µm, the ring‐like structures exhibit issues such as low brightness in certain regions and fragmentation. This phenomenon likely arises because, as the diameter of the colloidal photonic crystals increases, the randomness in the self‐assembly of colloidal nanospheres leads to the formation of more non‐ordered close‐packed regions, resulting in corresponding dark areas. Therefore, in constructing PUF labels, it is advisable to control the diameter of the colloidal photonic crystals within the range of 45 to 70 µm, ensuring the quality of the PUF and improving the accuracy of the final anti‐counterfeiting system.

Due to the physically unclonable nature of the self‐assembly of colloidal nanospheres, the size and position of the resulting ring‐like structures also exhibit physically unclonable characteristics. As shown in Figure S14 (Supporting Information), multiple images of “Ring”‐type colloidal photonic crystals with diameters of ≈50 µm, but distinct central ring patterns are displayed. This variability lays the foundation for the subsequent construction of PUF (physically unclonable function) labels.

### 2.3 Performance of the PUFs

After obtaining multiple optical microscope images of “Ring”‐type colloidal photonic crystals, a corresponding PUF database can be established. Inspired by human iris recognition, a machine learning network is constructed using an iris‐like algorithm to train and enhance the AI's recognition capability, thereby further improving the anti‐counterfeiting level. Figure S15 (Supporting Information) illustrates the (PUF code processing) steps of the iris‐like algorithm applied to a single optical microscope image of a “Ring”‐type colloidal photonic crystal (Figures S16–S22 (Supporting Information) present the step‐by‐step processing results for 18 such images).

**Figure 4:**
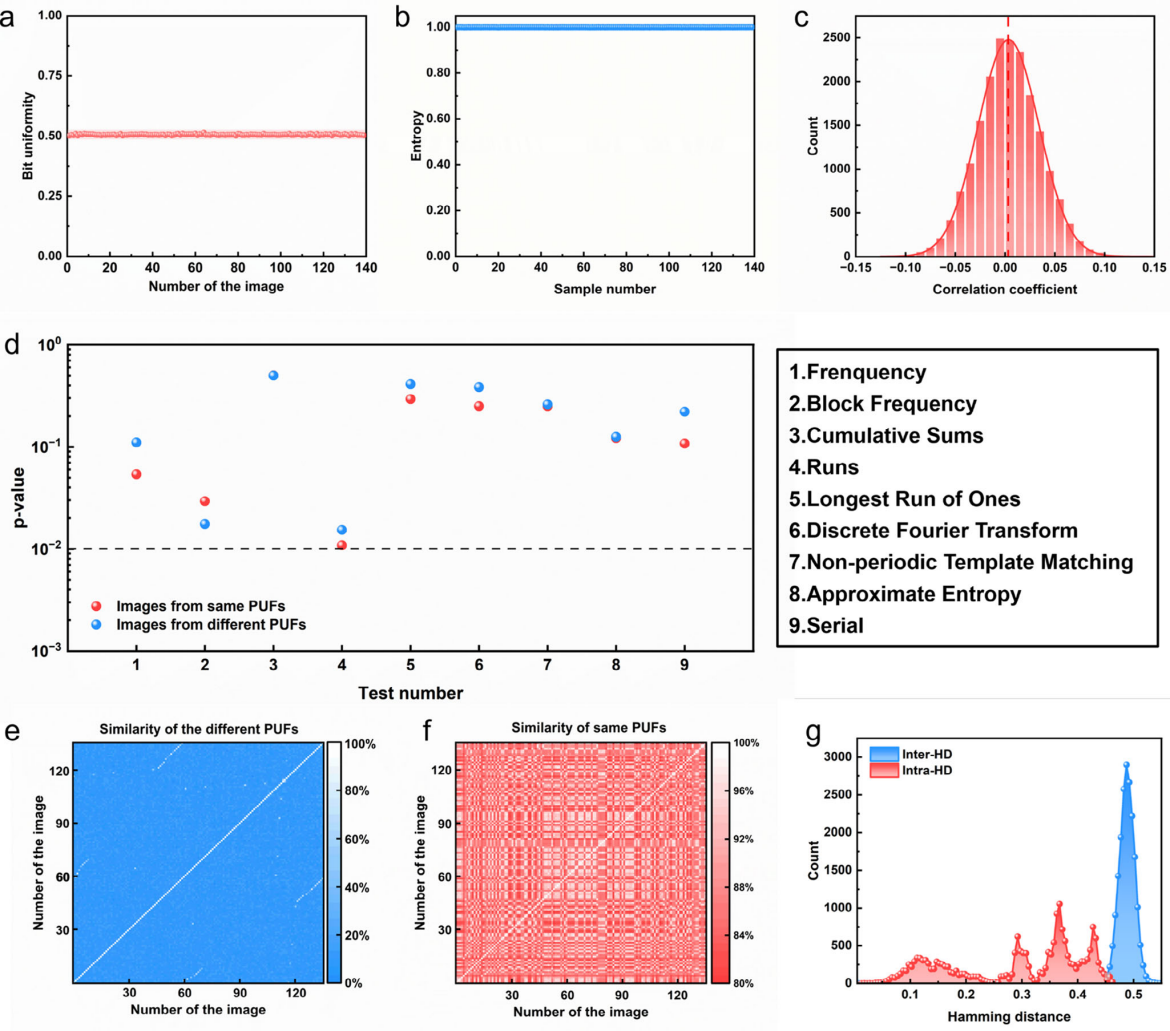
Randomness Evaluation of PUF Encoding a) Randomness evaluation using bit uniformity; b) Randomness evaluation using information entropy; c) Randomness evaluation using correlation coefficients; d) Randomness testing based on the National Institute of Standards and Technology (NIST) standards; e) Correlation heatmap of pairwise matching for images collected from different locations after encoding; f) Correlation heatmap of pairwise matching for images collected from the same location after encoding; g) Distribution plots of Inter‐HD and Intra‐HD.

To quantitatively evaluate the anti‐counterfeiting performance of PUF, the characteristics of PUF are analyzed. In a single PUF, the ideal scenario is that each pixel has an equal probability of being in the 0 or 1 state, which can be characterized by bit uniformity, information entropy, correlation coefficient, and NIST tests (Text S4 and Equations S9–S11, Supporting Information). **Figure** **4**a illustrates the probability distribution of “1” occurrences with the bit uniformity approaching the ideal value of 0.5. The emergence of the information entropy approaches the ideal value of 1 (Figure 4b), and the correlation coefficient approaches the ideal value of 0 (Figure 4c). Additionally, the industry‐standard NIST test (Supporting information Text 4.4) is introduced, and it is found that the p‐value for each test is greater than 0.01 (Figure 4d). They all indicate that the PUF encoding exhibits good randomness.

Subsequently, the Inter‐PUF group (139 images of PUF codes) derived from different “Ring”‐type colloidal photonic crystal images is characterized by the correlation coefficient, as shown in Figure 4e. It is observed that the similarity between different PUF codes is consistently below 10%, indicating that each corresponding PUF code is unique. The Intra‐PUF group (captured at different time and followed by the same coding procedure) is also characterized by the correlation coefficient, as shown in Figure 4f. It is observed that the similarity of the same PUF codes exceeds 80%. This demonstrates that each corresponding PUF code exhibits good robustness and reproducibility.

In order to more intuitively illustrate the relationship between Inter‐PUF group and Intra‐PUF group, we introduced the Hamming Distance, which is used to measure the similarity and differences among these PUF groups (Text S4 and Equation S12, Supporting Information). The Inter‐HD of Inter‐PUF group and the Intra‐HD of Intra‐PUF group are measured, as illustrated in Figure 4g. The Inter‐HD, representing the variation between different PUFs, follows a Gaussian distribution centered at 0.4875, which is close to the optimal value of 0.5. This indicates that all PUF codes exhibit significant randomness. The Intra‐HD, representing the variation within identical PUFs, shows a broader distribution but minimal overlap with the Inter‐HD. Among nearly 20 000 distributions, only 80 results in false acceptance rate (FAR) and false rejection rate (FRR) errors, yielding an error rate of ≈0.4% (Figure S23, Supporting Information). This meets the fundamental criteria for mutual distinguishability. Therefore, the aforementioned PUF codes possess strong randomness and can be effectively utilized for authentication in PUF‐based anti‐counterfeiting systems.

### 2.4 Authentication of the PUFs

**Figure 5:**
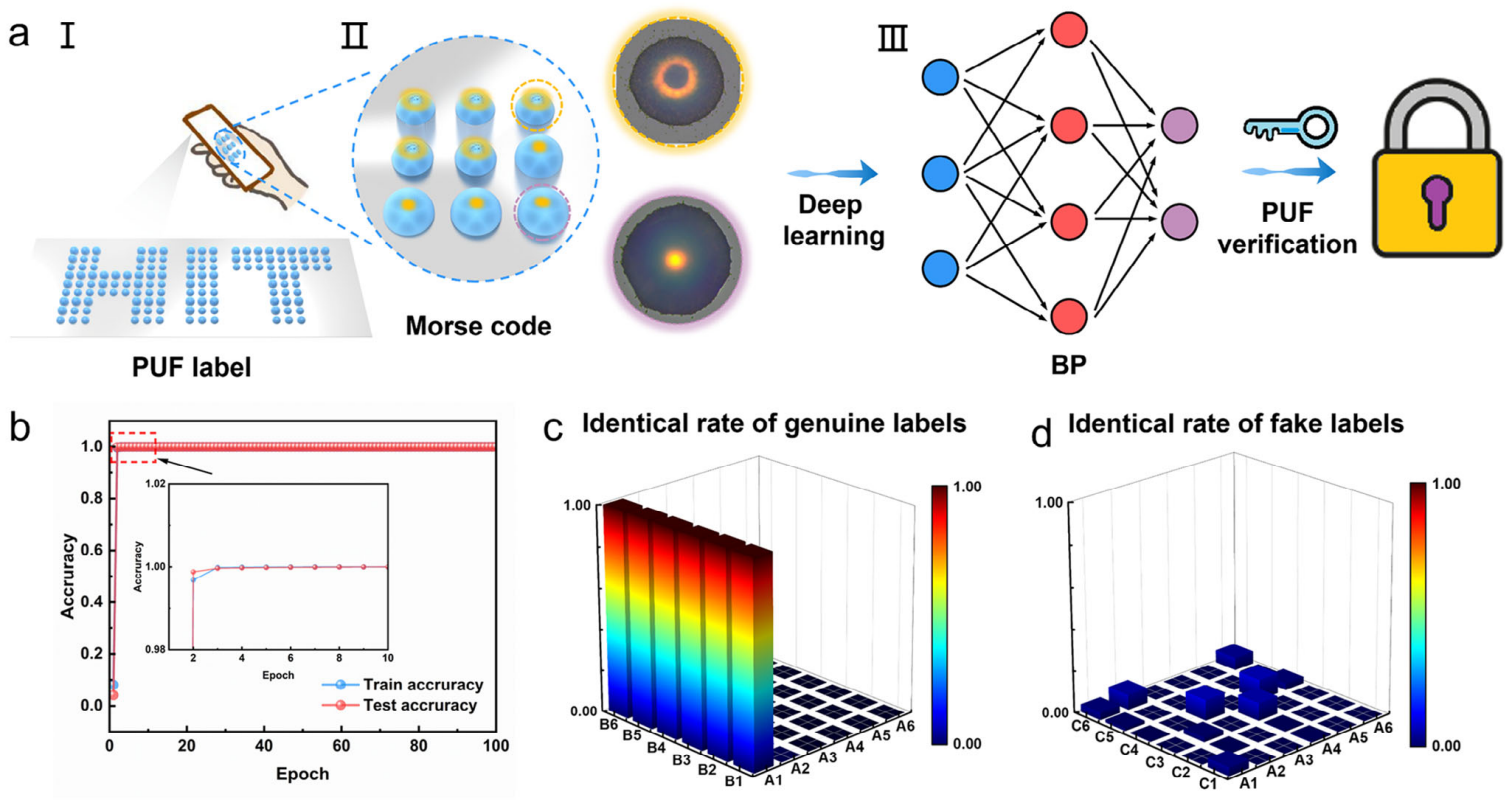
Optical PUF Multi‐layer Anti‐counterfeiting System Authentication Process Based on Structural Colors. a) The authentication process of the optical multi‐layer anti‐counterfeiting system, including macroscopic structural color patterns, microscopic "Morse codes," and the PUF iris structure; b) The machine learning cycle process; c) The machine learning matching results for labels B1‐B6 in the database, where B1‐B6 are derived from A1 (genuine); d) The machine learning matching results for labels C1‐C6 in the database, where C1‐C6 are not from the database (counterfeit).

For consumers, possessing a conspicuous and visually observable anti‐counterfeiting pattern is the most convenient anti‐counterfeiting method. Therefore, the structural color pattern constitutes the first layer of the anti‐counterfeiting system. This pattern, as shown in **Figure** **5**a‐I, an anti‐counterfeiting pattern featuring the “HIT” character is designed for the product. The “HIT” character is composed of a pixel array of colloidal photonic crystals, which can be further categorized into two types of pixels: “Ring”‐type colloidal photonic crystals and “Dot”‐type colloidal photonic crystals. These “Ring”‐type and “Dot”‐type colloidal photonic crystals are randomly arranged within the pattern. This arrangement can be achieved by controlling the printing process through a computer program in the electrohydrodynamic printing device.

Moreover, single‐layer structural color patterns are often prone to counterfeiting. Thus, the colloidal photonic crystal pixels within the “HIT” character are randomly distributed, with “Ring”‐type colloidal photonic crystals encoded as “1” and “Dot”‐type colloidal photonic crystals encoded as “0”, like the “Morse code” (Figure 5a‐II). The “Morse code” embedded within the structural color pattern constitutes the second layer of the anti‐counterfeiting system. Using the corresponding “1” and “0” values, a unique “Morse code” can be constructed (Figure S24, Supporting Information). Each “HIT” character pattern possesses a unique “Morse code”. The introduction of the “Morse code” not only enhances the overall anti‐counterfeiting level of the structural color pattern but also allows manufacturers to increase the encoding capacity of the “production number” by varying the size of the pixels and the structural color pattern. Taking a 1 cm × 1 cm structural color pattern as an example, if a colloidal photonic crystal array with a diameter of 50 µm and a spacing of 150 µm is constructed, an encoding capacity of 2^2500^ can be achieved, demonstrating excellent encoding performance.

Finally, based on the physically unclonable properties observed during the evaporation self‐assembly process of colloidal nanospheres, a third layer of the anti‐counterfeiting system is constructed. This involves the localization, normalization encoding, neural network learning, and recognition authentication of the ring structures within the “Ring”‐type colloidal photonic crystals, as illustrated in Figure 5a‐III.

Through the structural color pattern, the “Morse code” composed of “Ring”‐type and “Dot”‐type colloidal photonic crystals, and the PUF code generated by the ring structures of “Ring”‐type colloidal photonic crystals, a multi‐layer optical PUF anti‐counterfeiting system is constructed. The workflow is as follows: First, the manufacturer produces the corresponding optical PUF labels. Then, the manufacturer records the “Morse code” of the randomly generated PUF labels composed of “Ring”‐type and “Dot”‐type colloidal photonic crystals, and compiles an image dataset from the “Ring”‐type colloidal photonic crystal images, which is then provided to a neural network model for training. The trained neural network model is subsequently used for authenticity verification. When consumers receive the product, they can perform an initial verification using the “production code” provided by the manufacturer, then upload the “Ring” structure pattern image to the system provided by the manufacturer. The trained neural network model can then determine whether the product is “Genuine” or “Fake” based on the pattern information uploaded by the consumers.

To further illustrate the verification process of the optical PUF anti‐counterfeiting system, as shown in Figure S25 (Supporting Information), six images of “Ring”‐type colloidal photonic crystals are captured (Figure S25a‐I–a‐VI, Supporting Information). These images undergo image preprocessing, enhancement, and a series of feature extraction steps to construct a database for training the machine learning model. During the model training process, the database is randomly shuffled, with 80% of the data used for training and the remaining 20% for validation. The efficiency of feature extraction methods such as the Hough transform is utilized to enable the neural network model to rapidly fit the dataset, achieving accuracy close to 1 in both training and validation stages (Figure 5b). The verification of the six samples labeled as genuine is also entirely correct (Figure 5c). Subsequently, six additional images (b‐I‐b‐VI) are constructed, representing the images uploaded to the cloud by consumers after purchasing genuine products (a‐I). For further comparison, six counterfeit PUF patterns (not part of the database, Figure S25c‐I–c‐VI (Supporting Information) are fabricated and input into the anti‐counterfeiting system in the same manner. The output result shows a low matching possibility (Figure 5d), indicating a “Fake” judgment. This demonstrates that the anti‐counterfeiting system successfully identifies the “counterfeit” PUF labels.

In summary, through the structural color pattern, the “Morse code” embedded within the structural color pattern composed of “Ring”‐type and “Dot”‐type colloidal photonic crystals, and the optical PUF label constructed from the ring structure of “Ring”‐type colloidal photonic crystals, a multi‐layer anti‐counterfeiting system is developed, characterized by a low recognition threshold and excellent anti‐counterfeiting performance. The verification of the optical PUF label can be accomplished with a simple optical microscope. Compared with the Fluorescent PUF and Raman PUF, the equipment the system requires is simpler, more convenient, and faster. Therefore, our multi‐layered system represents a significant advancement in cost‐effective and scalable anti‐counterfeiting technologies.

## 3 Conclusion 

A multi‐layer optical anti‐counterfeiting system inspired by the structural color iris is proposed in this work. First, experimental and simulation results demonstrate that the ring structure in “Ring”‐type colloidal photonic crystals arises due to surface tension‐induced droplet reconstruction. As the contact angle decreases and the three‐phase contact line is pinned, the self‐assembly force of colloidal nanoparticles weakens, making it more difficult for them to accumulate toward the center. Consequently, an ordered ring‐like accumulation forms around the periphery. By tuning the intensity of the “coffee‐ring” effect, the transition between “Ring”‐type and “Dot”‐type colloidal photonic crystals can be controlled. Second, multiple characteristic parameters, including bit uniformity, information entropy, similarity analysis, NIST tests, intra‐HD, inter‐HD, FAR, and FRR, confirm that the PUF codes generated from “Ring”‐type colloidal photonic crystal images exhibit uniqueness and readability. Finally, a multi‐layer anti‐counterfeiting system with a low recognition threshold (spanning from macroscopic to microscopic levels) and excellent anti‐counterfeiting performance (0 false alarm rate) is constructed through three key components: i) the structural color pattern; ii) the embedded “Morse code” formed by “Ring”‐type and “Dot”‐type colloidal photonic crystals; and iii) the optical PUF labels derived from the “Ring”‐type colloidal photonic crystals. This system provides a cost‐effective solution for combating counterfeit products and further promotes the practical application of structural color technology in the anti‐counterfeiting of high‐value products.

## 4 Outlook

Current PUF security research is evolving from “physical anti‐counterfeiting” to “algorithm‐physical joint anti‐counterfeiting,” where the unique attributes of novel PUFs introduce new attack surfaces for adversaries. While our optical PUF multi‐layer anti‐counterfeiting system provides cost‐effective physical anti‐counterfeiting solutions, its algorithmic defense mechanisms need further improvement. For instance, Yang^[^ ^64^ ^]^ et al. developed a high‐entropy electromagnetic PUF using non‐Hermitian parity‐time symmetric structures, demonstrating robust resistance against AI‐driven attacks (e.g., Fourier regression and GANs) while achieving near‐ideal uniqueness and cross‐spectral scalability. Therefore, future work must integrate multi‐modal encoding strategies with machine learning attack‐resistant methods to establish an integrated “detection‐defense‐self‐healing” PUF security framework.

## 5 Experimental Section

### Materials

Potassium persulfate (KPS), sodium dodecylbenzenesulfonate (LAS), and sodium bicarbonate were purchased from Aladdin Biochemical Technology Co., Ltd. (Shanghai, China). Octadecyltrichlorosilane (OTS), cyclohexane, styrene, and α‐methylacrylic acid were obtained from Macklin Biochemical Technology Co., Ltd. (Shanghai, China). All reagents were of analytical grade and did not require further purification prior to use. Silicon wafers were purchased from Lijing Electronics Co., Ltd. (Zhejiang, China). Ultrapure water was prepared using a Milli‐Q water purification system (resistivity > 18 MΩ·cm).

### Characterization

The synthesized PS‐COOH particles and the hemispherical microdomains of colloidal photonic crystals formed via evaporation‐induced self‐assembly were observed using a field emission scanning electron microscope (Gemini 560, Zeiss, Germany). The particle size and surface potential of the synthesized PS‐COOH were measured using a Malvern laser particle size analyzer (Zetasizer Nano ZSP, Malvern, UK). The electrohydrodynamic printing process was recorded using a high‐speed camera (MINIUX100‐C‐32GB, China). The contact angle of droplets on silicon wafer substrates was measured using a contact angle goniometer (JC2000, China). The organic functional groups of PS‐COOH were characterized using Fourier transform infrared spectroscopy (Nicolet iS50, Thermo Fisher, USA). The hemispherical microdomains of colloidal photonic crystals were observed using a reflection optical microscope (DM4 M, Leica, Germany). The reflection spectra of the unit structures of colloidal photonic crystals were tested using a reflection optical microscope (iM1, Olympus, Japan) equipped with a micro‐area reflection spectrometer (NOVA, Ideaoptics, China). The cross‐sectional morphology of the colloidal photonic crystals was observed using a dual‐beam scanning electron microscope (Crossbeam 350, Zeiss, Germany). The hemispherical microdomains of colloidal photonic crystals were characterized using Raman spectroscopy (Horiba LabRAM HR Evolution, Japan).

### Synthesis of PS‐COOH Nanospheres

PS‐COOH nanospheres were synthesized via emulsion polymerization. Prior to the reaction, the styrene monomer was repeatedly washed three times using a solid‐phase extraction column containing basic alumina to remove the inhibitor from the raw material. Before the reaction begins, 60 mL of ultrapure water, 0.1 mL of α‐methacrylic acid, 13.2 mg of LAS, and 28.2 mg of NaHCO3 were added to a three‐necked flask. A magnetic stirrer was placed in the flask, and the three necks were connected to a nitrogen gas inlet, a condenser reflux tube, and a gas‐washing bottle to prevent backflow, respectively. The stirring speed was set to 300 r min^−1^, and the temperature was raised to 60 °C. Then, 5.5 mL of the styrene monomer was added, and nitrogen gas was introduced for 30 min to remove oxygen from the apparatus. Subsequently, 1 mL of a 0.5 mg mL^−1^ potassium persulfate (KPS) solution was added, and the reaction was carried out at 70 °C under nitrogen protection for 8 h. Finally, the white emulsion obtained from the reaction was centrifuged at 13 000 rpm for 20 min, and a clear color pattern could be observed on the walls of the centrifuge tube. The resulting precipitate was washed three times with deionized water and then dispersed and stored in water by shaking.

### Hydrophobic Treatment of Silicon Wafer Substrates

Initially, a piranha solution was prepared. The polished silicon wafers, after being cut, were immersed in this solution for 10 s to remove any residual organic impurities on the surface. Subsequently, the wafers were thoroughly rinsed with deionized water and dried using high‐purity nitrogen gas, then placed in a petri dish for further use. The prepared silicon wafers were then placed in a plasma treatment chamber, where oxygen was introduced. The power was set to 100 W, and the treatment lasts for 300 s. At this stage, the silicon wafer surfaces were endowed with a substantial number of hydroxyl groups. Following this, the wafers were placed in a glass petri dish (plastic petri dishes should not be used), and a mixture of cyclohexane and OTS (with a volume ratio of 1:100) was added. The wafers were left to stand at room temperature for 24 h, ensuring that they do not overlap as much as possible. After this period, the wafers were removed and subjected to ultrasonic treatment in a tetrahydrofuran solution for 15 min to eliminate unreacted alkyl groups and hydrolysis byproducts. Finally, the wafers were rinsed three times with ethanol and deionized water, wiped with dust‐free paper, and stored in a petri dish for subsequent use.

### Electrohydrodynamic Inkjet Patterning

The colloidal photonic crystal hemispherical microdome structural color patterns were printed using an electrohydrodynamic inkjet printing device. Approximately 1 mL of colloidal photonic crystal ink was drawn into a precision capillary glass tube (diameter 45 µm) using a syringe. The capillary glass tube was then fixed, and a copper wire was connected to the positive electrode of a high‐voltage power supply. The distance between the capillary glass tube and the substrate was adjusted to an appropriate value (∼ 0.15 mm), and the voltage was applied for printing (Figure S3, Supporting Information). Finally, the printed silicon wafer was placed in a constant temperature and humidity chamber at 30 °C with a relative humidity (RH) of 60% for evaporation‐induced self‐assembly.

### Simulation of Reflection Spectral Intensity

The reflection spectra of colloidal photonic crystals were calculated using the commercial finite element software Lumerical FDTD Solution. In the software, a face‐centered cubic (FCC) photonic crystal close‐packed structure with the (111) plane located on the X‐Y plane was first constructed to simulate the ordered layer of the colloidal photonic crystal. Since disordered regions were prone to form during the self‐assembly process of colloidal nanospheres, disordered regions were also introduced as a supplement. The Object Library module in the software was used to introduce a Random particles region to simulate the disordered regions in the colloidal photonic crystal. The colloidal nanospheres in both the disordered and ordered regions have the same diameter and refractive index, differing only in their arrangement. In the model, a plane wave (Plane source) was used as the light source, with a wavelength range of 400–760 nm, incident vertically from the Z‐axis onto the (111) close‐packed plane. Above the light source, a frequency domain field profile monitor was set up to capture the reflected light data and obtain the reflection spectrum. To ensure the resulting curve was sufficiently smooth, 100 frequency points were set. In the calculations, the refractive index of the polystyrene colloidal nanospheres was set to 1.587. To optimize the calculation results, an auto non‐uniform mesh was selected, with a mesh accuracy of 4 and a mesh size of 10 nm, which was much smaller than the size of the colloidal nanospheres constituting the photonic crystal. The time step stability factor (dt stability factor) was set to 0.2 to ensure convergence. Additionally, to avoid interference caused by the light source under periodic boundary conditions, which could lead to non‐convergence, the boundary conditions of the simulation region in the X, Y, and Z directions were set as perfectly matched layers (PML), with 18 layers configured.

### Machine Learning and Verification Methods

The neural network employed in this study was created using the patternnet function in MATLAB software, with the number of hidden neurons set to 200. To obtain a sufficiently large training dataset, image augmentation was performed on the originally acquired images of “Ring”‐type colloidal photonic crystals. By applying slight rotations, 99 additional images were generated from each original image. Subsequently, the bionic iris structures in these images were localized, segmented, and normalized to construct the dataset. Eighty percent of this dataset was used for training, while the remaining 20% was reserved for validation. The training process was configured with 100 epochs and a learning rate of 0.01.

In practical applications, when consumers submit randomly captured images of genuine anti‐counterfeiting labels using a fluorescence microscope, the system automatically retrieves the corresponding accurate information and provides a detailed matching score with the indexed reference. For images that have not been previously learned, such as those from counterfeit products, the artificial intelligence assigns a lower matching score. Operating as a black box, the deep learning system enables consumers to verify the authenticity of products containing PUF security labels without requiring knowledge of the underlying mechanisms.

## Conflict of Interest

The authors declare no conflict of interest.

## Supporting information



Supporting Information

Supporting Information

Supporting Information

## Data Availability

The data that support the findings of this study are available in the supplementary material of this article.
