# Supplementary material for: Bio‐Inspired Structural Color Irises as Physical Unclonable Functions for Anti‐Counterfeiting
Source: Adv Sci (Weinh). 2025 Jul 10;12(34):e04771. doi: 10.1002/advs.202504771 (PMC12442702; doi:10.1002/advs.202504771)
Supplement: Supplementary file 1 — Supporting Information [file ADVS-12-e04771-s002.docx]

Supporting Information

**Bio-Inspired Structural Color Irises as Physical Unclonable Functions for Anti-Counterfeiting**

*Kongyu Ge^a+^, Yiming Hu^b+^, Jiangnan Yan^a^, Jianing Ding^a^, Yifan Gao^a^, Hongyu Yi^a^, Zhan Li^a^, Lingfei Cui* *^d^, Shaowei Hu^c^, Hongjun Ji^c^, Mingyu Li^c^, Ruobing Bai* *^d^*, Rongpei Shi^b^* and Huanhuan Feng^a^**

*^a^* Sauvage Laboratory for Smart Materials, Shenzhen Key Laboratory of Flexible Printed Electronics Technology, Harbin Institute of Technology (Shenzhen),

Shenzhen 518000 (China)

E-mail: [fenghuanhuan@hit.edu.cn](mailto:fenghuanhuan@hit.edu.cn)

*^b^* Harbin Institute of Technology (Shenzhen)

Shenzhen 518000 (China)

E-mail: shirongpei@hit.edu.cn

*^c^* State Key Laboratory of Advanced Welding and Joining (Shenzhen), Harbin Institute of Technology (Shenzhen)

Shenzhen 518000 (China)

^d^ School of Mechanical Engineering, Shanghai Jiao Tong University, Shanghai 200240, China

E-mail: bairuobing@sjtu.edu.cn

^+^ These authors contributed equally to this work

^*^ Corresponding authors


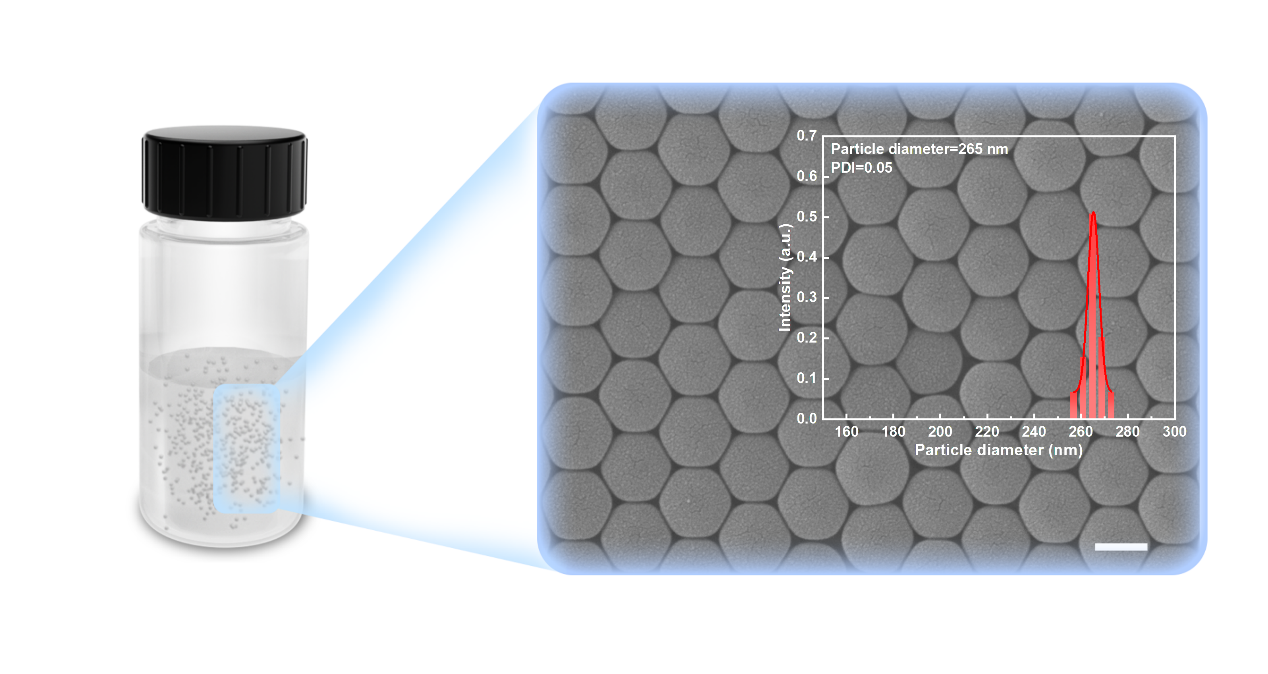


Figure S1 PS-COOH colloidal photonic crystal ink (the right panel shows the SEM image with a scale bar of 200 nm; the inset illustrates the particle size distribution).


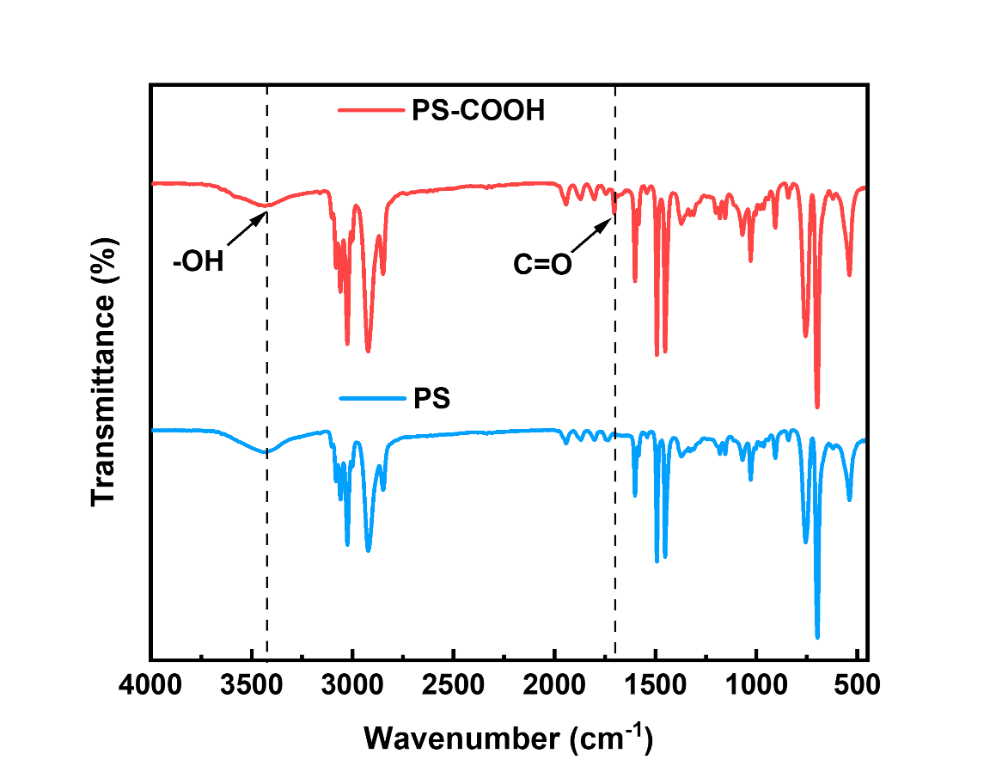


Figure S2 Comparison of FTIR spectra between PS-COOH and PS.


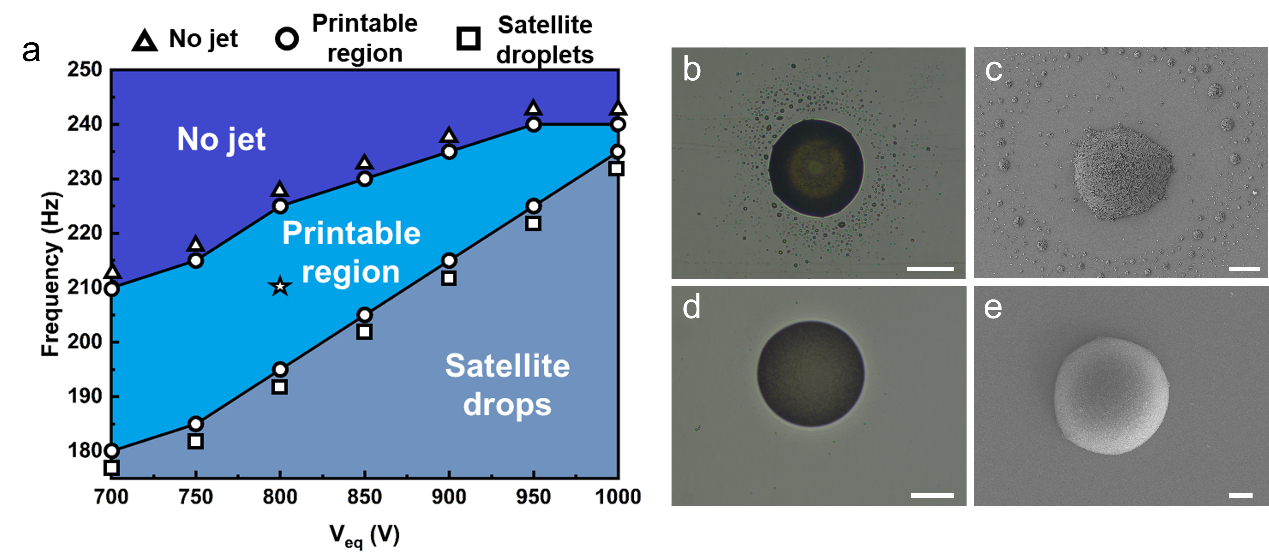


Figure S3 Electrohydrodynamic printing patterns

(a) Phase diagram of electrohydrodynamic printing; (b-c) Optical micrograph and SEM image of satellite droplet regions, with scale bars of 50 μm and 10 μm, respectively; (d-e) Optical micrograph and SEM image of printable regions, with scale bars of 50 μm and 10 μm, respectively.

The printed phase diagram of electrohydrodynamic printing is plotted by varying the frequency of the pulsed square wave and the equivalent voltage V_eq_ (with the bias voltage and the amplitude voltage maintained at equal values). As illustrated in Figure S3, the phase diagram comprises three distinct regions: a no-jet region, a printable region, and a satellite droplet region. Notably, the upper critical frequency of the stabilized printable region increases from 210 to 240 Hz, while the lower critical frequency rises from 180 to 235 Hz, with the voltage increasing from 700 to 1000 V. Furthermore, the printable region shrinks as it moves upward with the voltage increase. This phenomenon is attributed to the nonlinear distribution of the electric field between the nozzle tip and the substrate.

Furthermore, when the frequency of the pulse voltage exceeds the upper critical frequency (as indicated by the hollow triangles in Figure S3 (a)), the colloidal photonic crystal ink fails to eject into the no-jet region. This occurrence may be attributed to the rapid frequency of the pulse voltage, causing the relaxation of the fluid to impede droplet breakage caused by the surface tension. Consequently, it is imperative to maintain the pulse voltage frequency below the upper critical frequency to guarantee drop ejection. Conversely, when the pulse voltage frequency is lower than the lower critical frequency (indicated by the hollow square in Figure S3 (a)), satellite drops emerge around the main drop and enter the satellite drop region. This phenomenon may result from insufficient pulse voltage frequency, resulting in poor drop continuity at the tip of the Taylor cone, thereby generating satellite drops, as illustrated in Figure S3 (b). The presence of satellite droplets impacts the surrounding droplets, diminishing the resolution of the evaporated self-assembly pattern, as illustrated in Figure S3 (c). Consequently, it is essential to maintain a pulse voltage frequency above the lower critical frequency to ensure the formation of a single stabilized drop (Figure S3 (d) and S3 (e)). Owing to the nonlinear nature of the stabilized printing process window, the pulse voltage parameter stabilizes the center of the printable region (shown as a hollow pentagram in Figure S3 (a)).


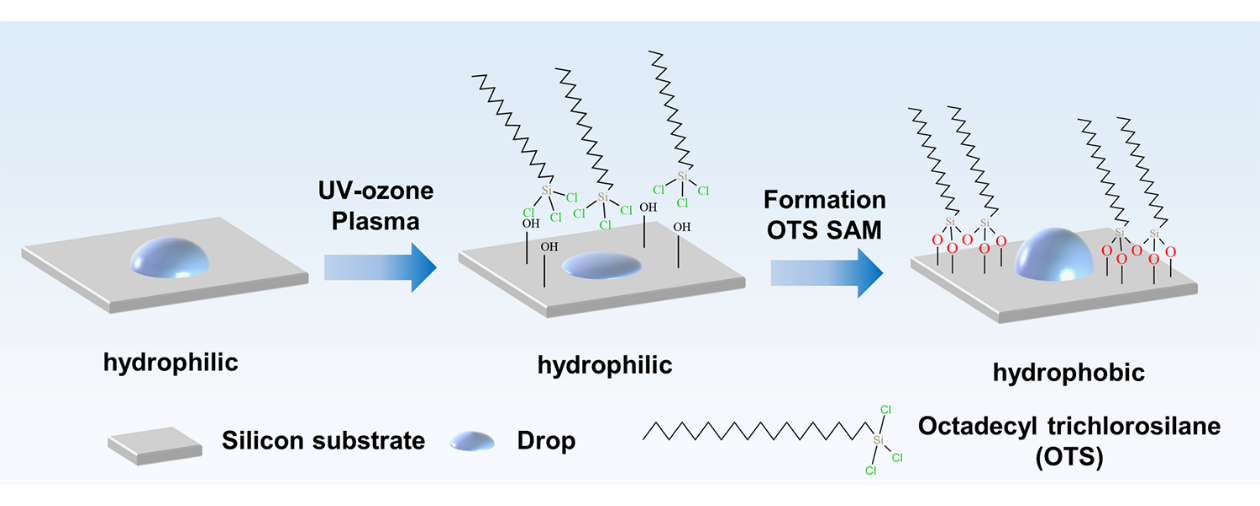


Figure S4 Schematic illustration of the hydrophobic substrate treatment process.

First, the silicon wafer substrate is cleaned, and the drop contact angle reaches approximately 80°. Then, the cleaned silicon wafer undergoes plasma treatment, which introduces hydrophilic –OH groups, reducing the contact angle to around 62°. Finally, treatment with silane reagent (OTS) introduces hydrophobic alkyl groups, increasing the contact angle to approximately 105°. This process yields substrates with three distinct contact angles. (For detailed procedures, please refer to Section "4.4 Hydrophobic Treatment of Silicon Wafer Substrates")


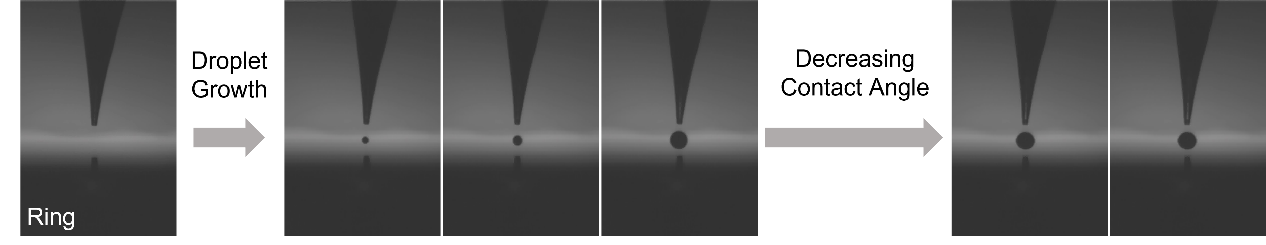


Figure S5 Real-Time Images of the "Ring"-Type Droplet Printing Process


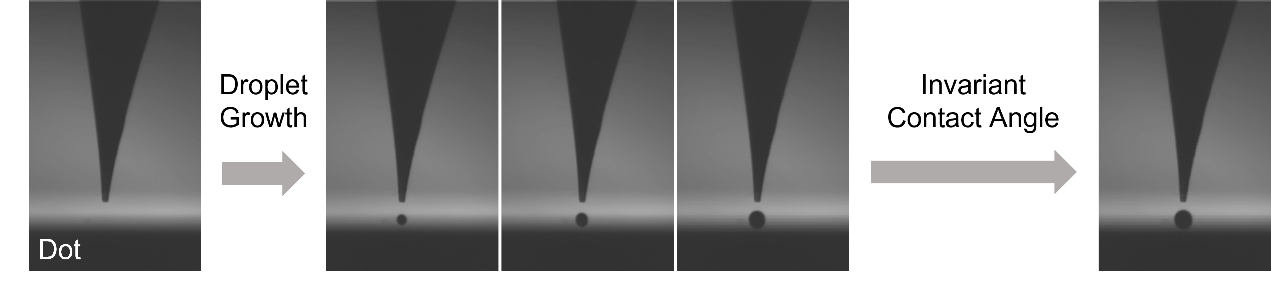


Figure S6 Real-Time Images of the "Dot"-Type Droplet Printing Process


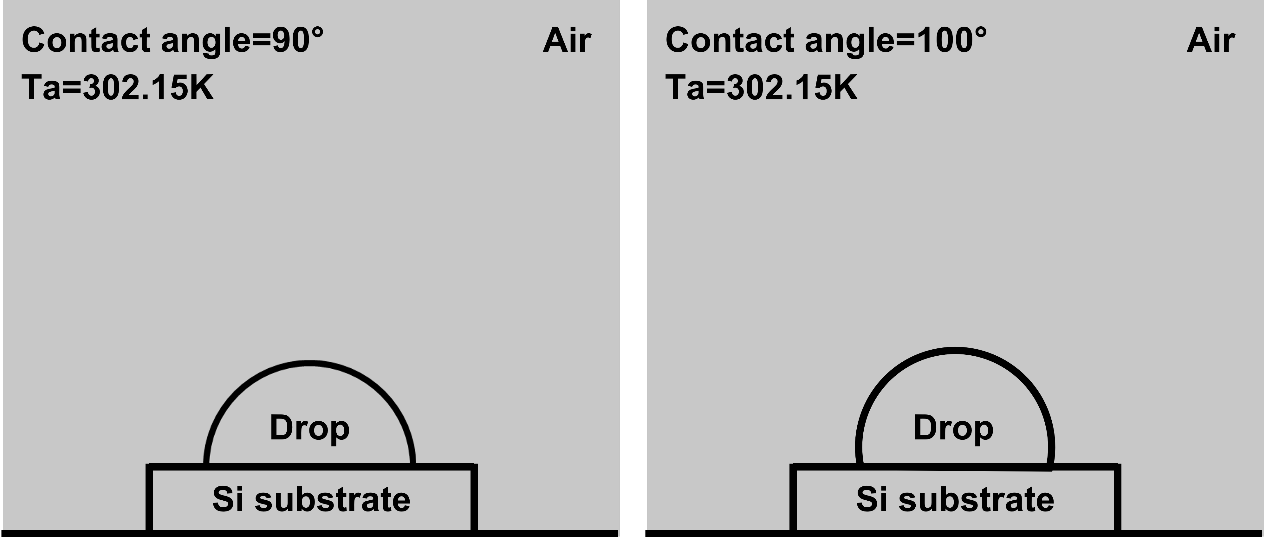


Figure S7 "Ring"-Type and "Dot"-Type Droplets Modeling Schematic


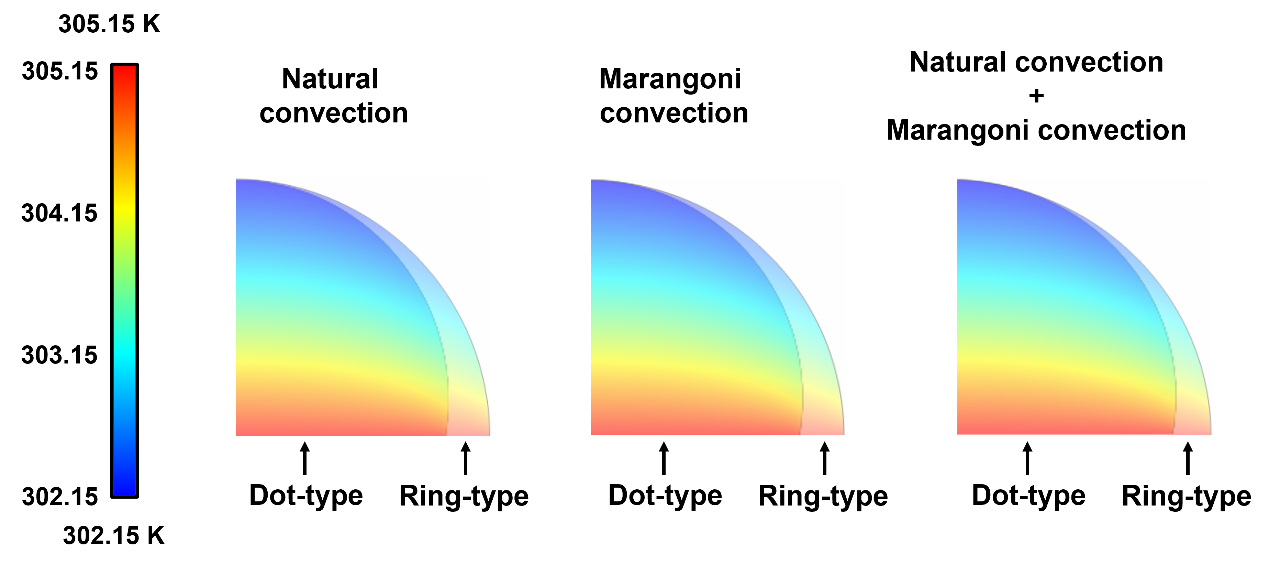


Figure S8 "Ring"-Type and "Dot"-Type Droplet Temperature Simulation Schematic


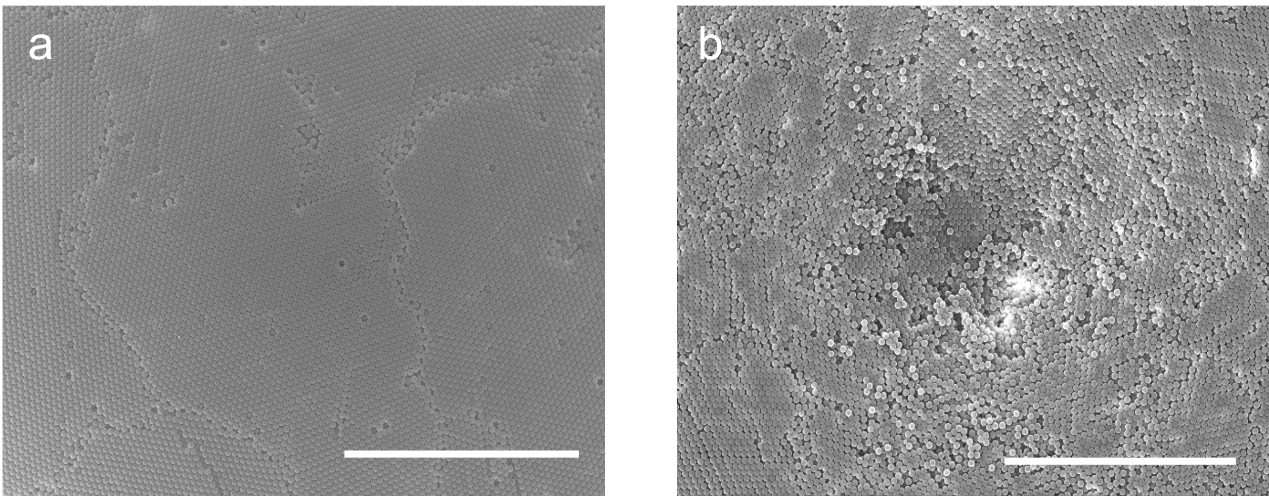


Figure S9 Top-View SEM Images of "Dot"-Type and "Ring"-Type Colloidal Photonic Crystals.

(a) Top-view SEM image of the "Dot"-type colloidal photonic crystal, with a scale bar of 10 μm; (b) Top-view SEM image of the "Ring"-type colloidal photonic crystal, with a scale bar of 10 μm.


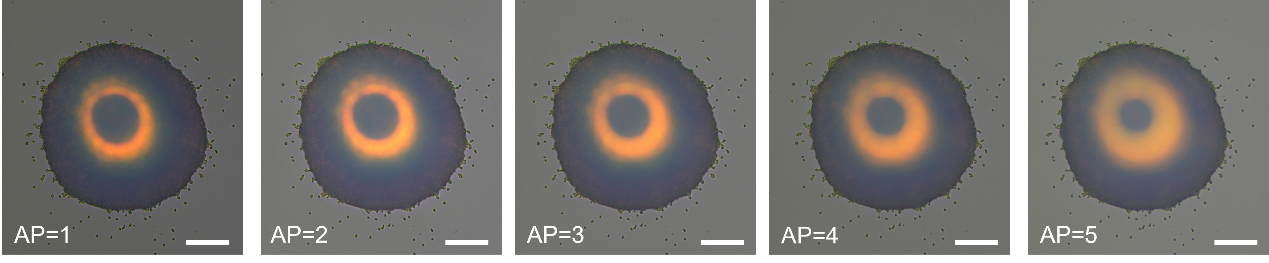


Figure S10 Optical micrographs of "Ring"-type colloidal photonic crystals under different aperture angles (AP) with a scale bar of 20 μm.

With the increase of the AP value, the halo in the "Ring"-type colloidal photonic crystals gradually spreads. However, there is no obvious offset or movement in the position of the halo centerline. This indicates that the generation of the halo structure comes from the surface of the "Ring"-type colloidal photonic crystal, where the colloidal nano-microspheres are arranged in an ordered annular manner.


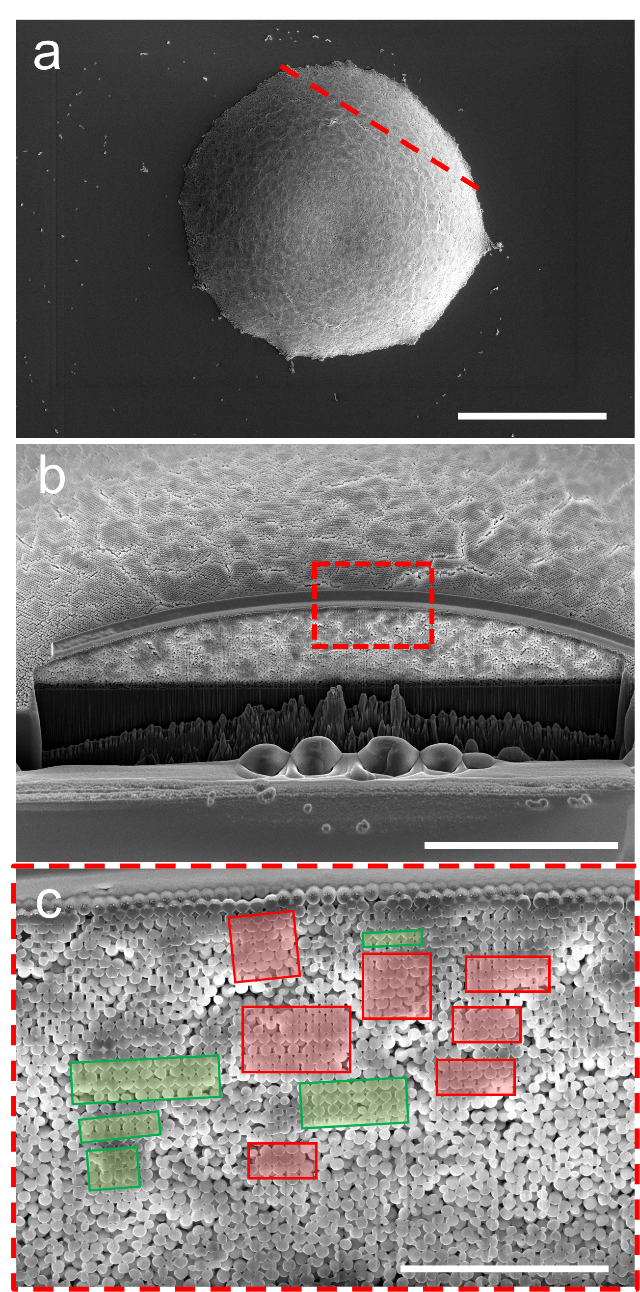


Figure S11 Cross-sectional views of "Ring"-type colloidal photonic crystals

(a) Scanning electron microscopy (SEM) image of "Ring"-type colloidal photonic crystal with a scale bar of 50 μm; (b) Cross-sectional view of "Ring"-type colloidal photonic crystal with a scale bar of 20 μm; (c) Magnified cross-sectional view of the red dashed box in (b) with a scale bar of 4 μm


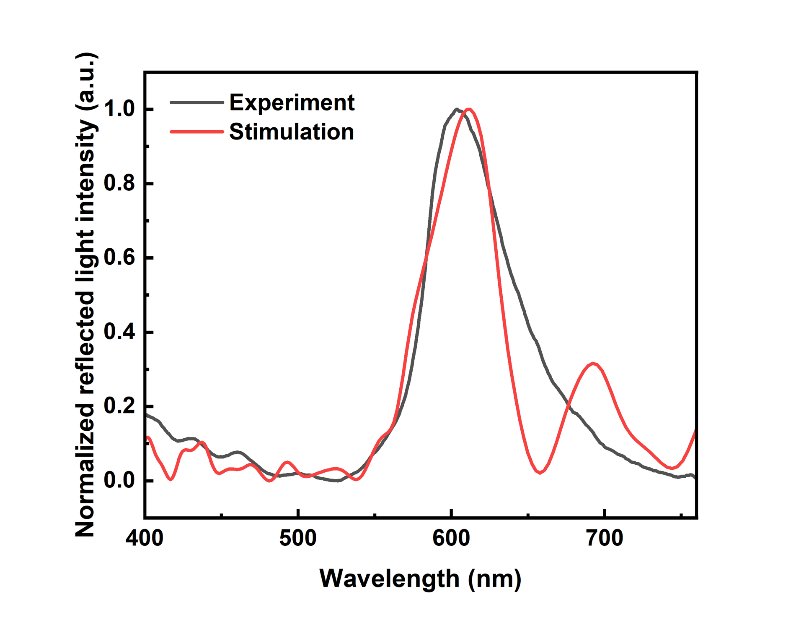


Figure S12 Comparison between simulated and experimentally measured reflectance spectra.


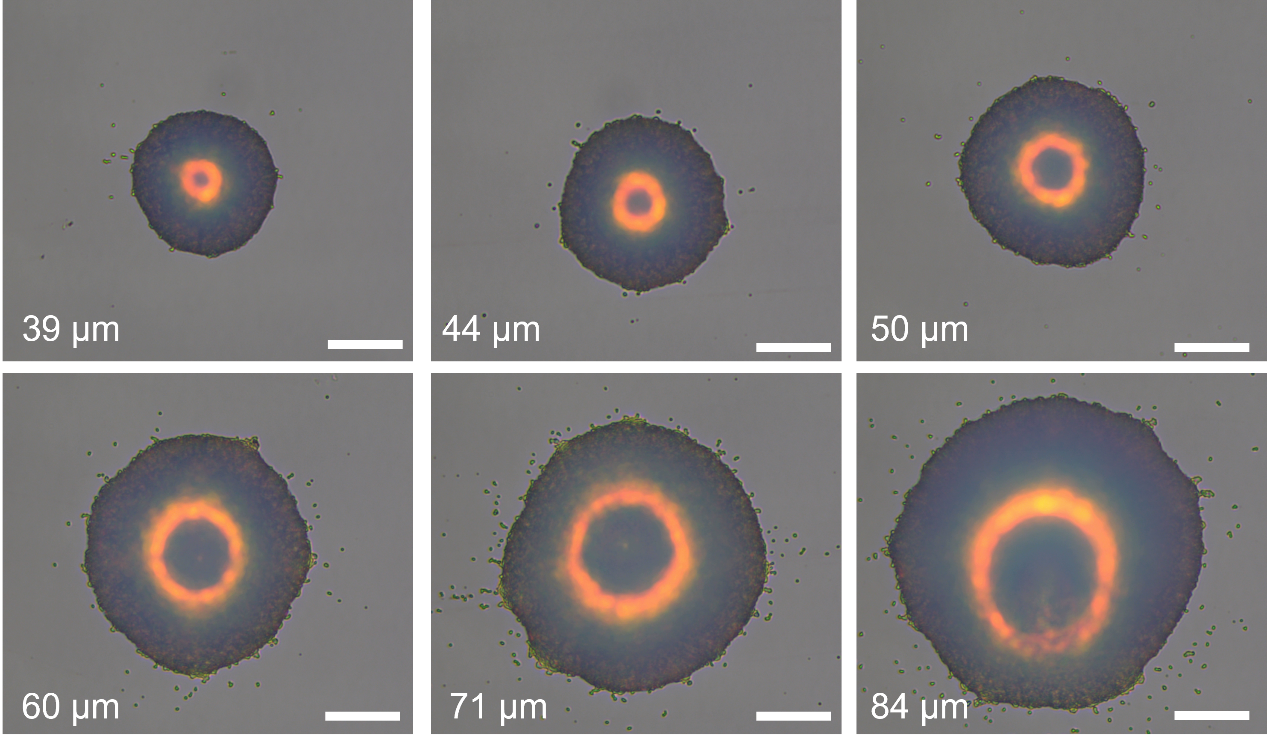


Figure S13 Optical micrographs of "Ring"-type colloidal photonic crystals with varying diameters. Scale bar: 20 μm


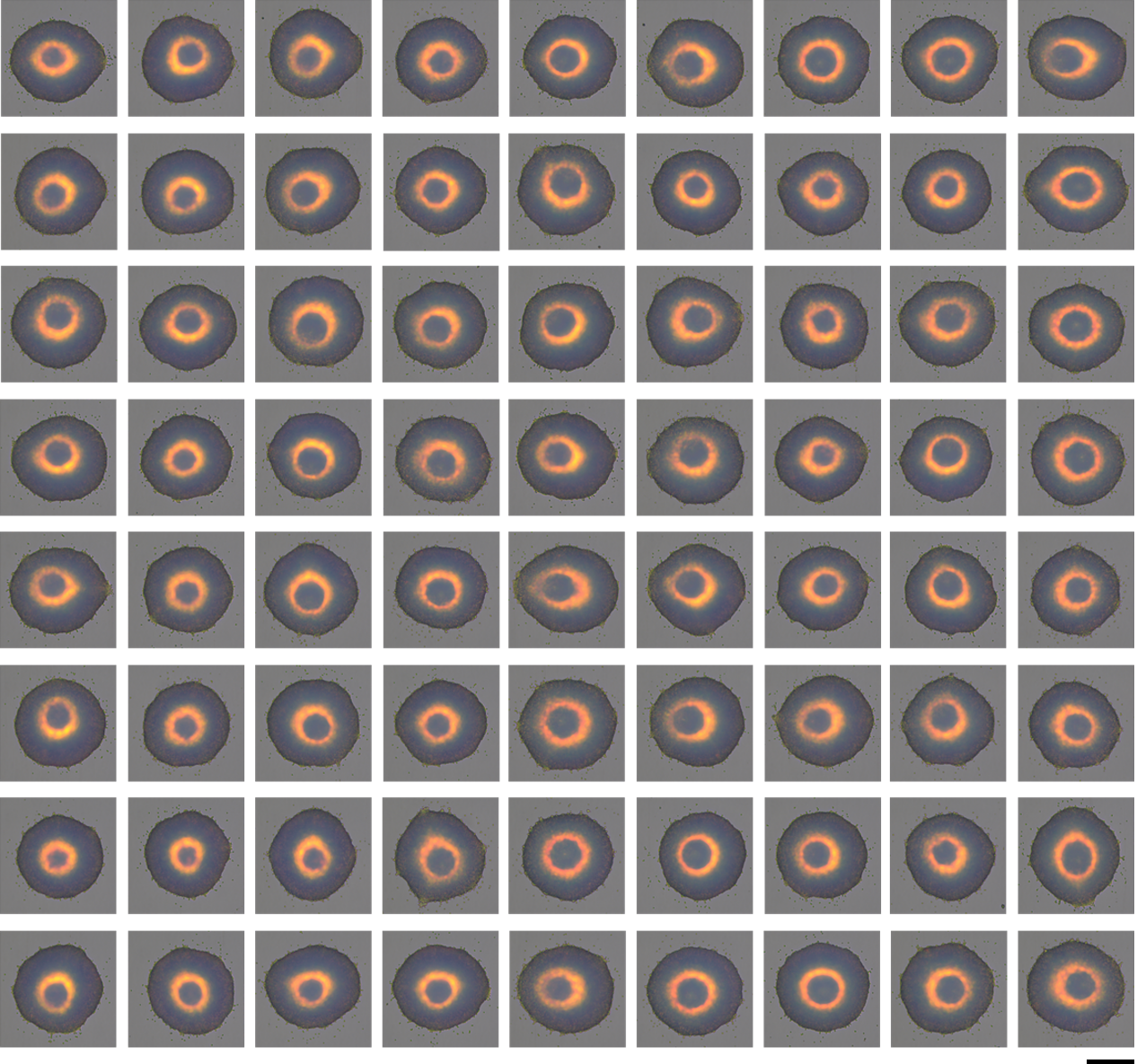


Figure S14 Selected 72 representative optical microscopy images of "Ring"-type colloidal photonic crystals, with a scale bar of 20 μm.


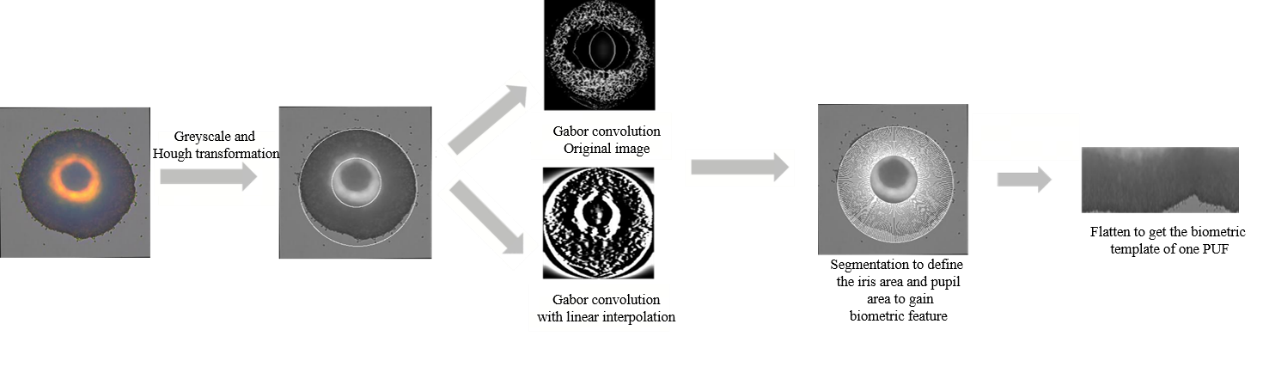


Figure S15 PUF encoding process based on hough transform


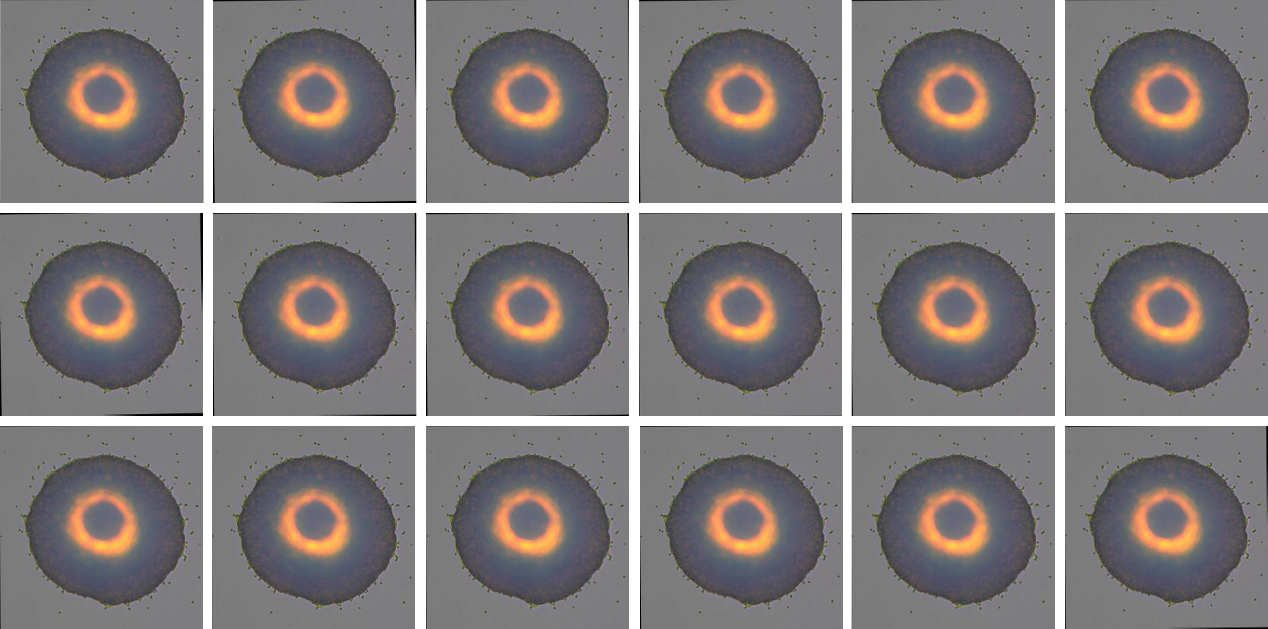


Figure S16 Original images after minor angular transformations


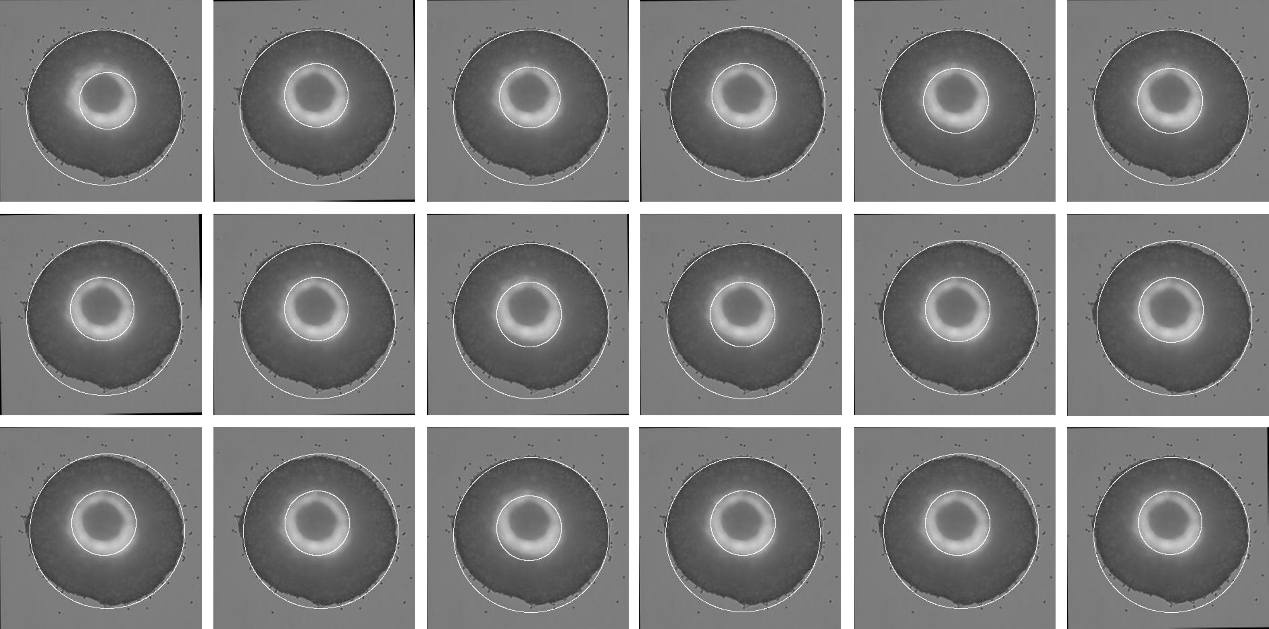


Figure S17 The inter and outer contours of the 18 images in Figure S22 are determined


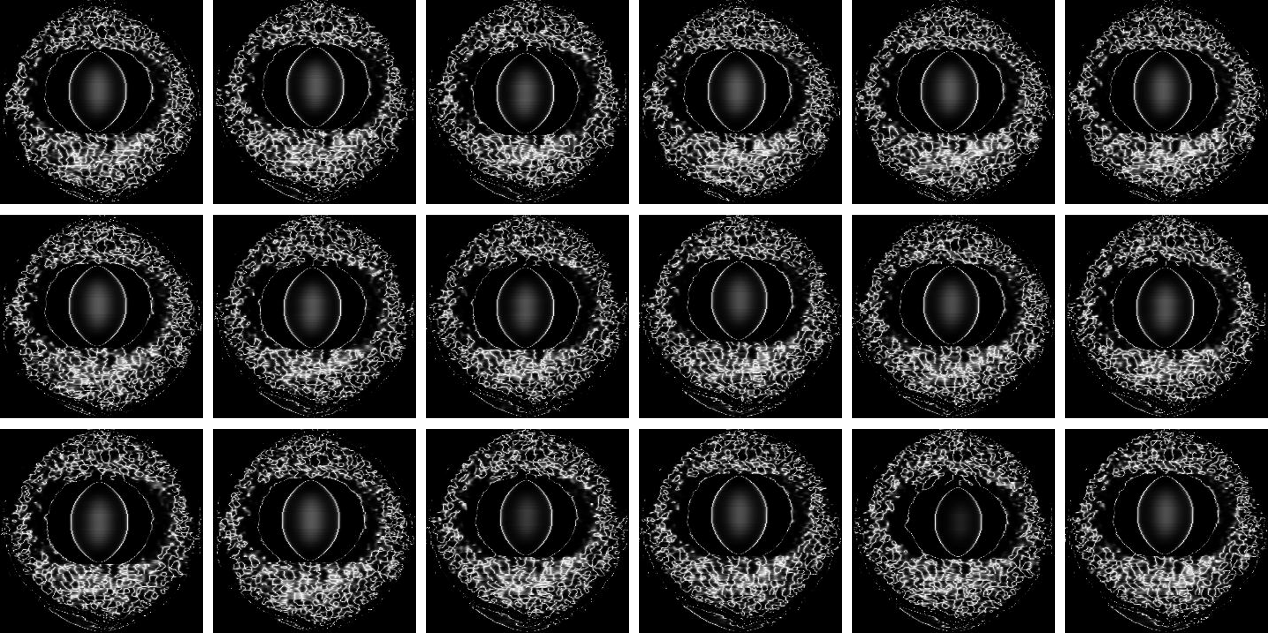


Figure S18 The outer contours of the 18 images in Figure S22 are determined through grayscale processing


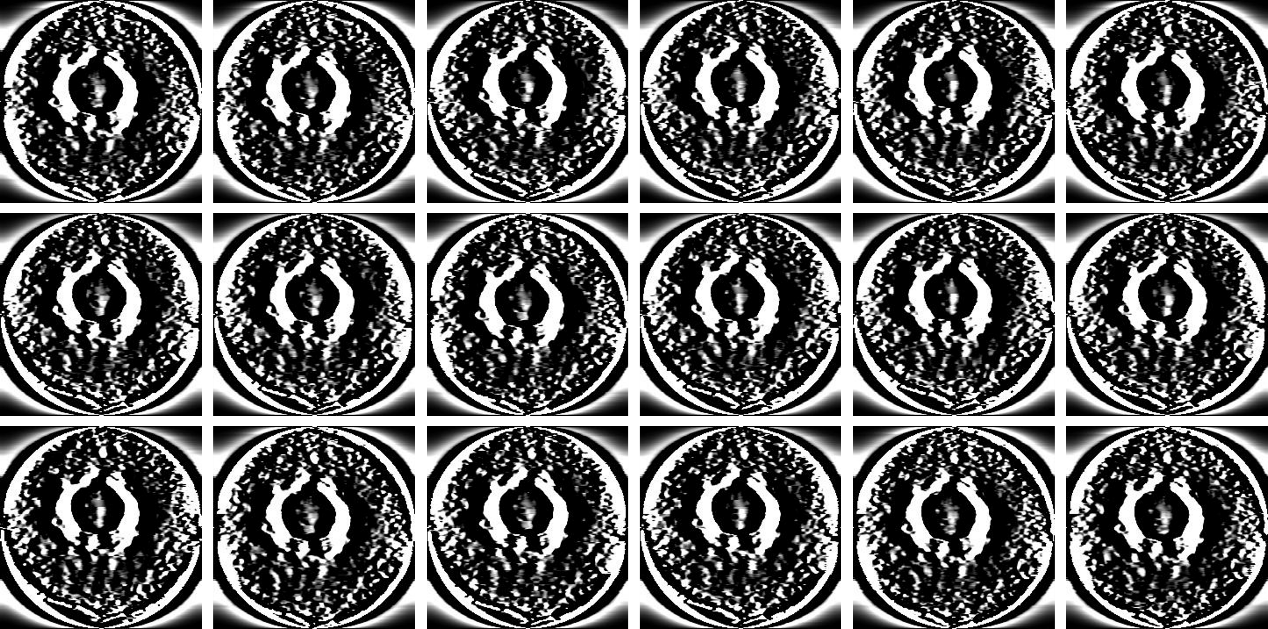


Figure S19 The inter contours of the 18 images in Figure S22 are determined through grayscale processing


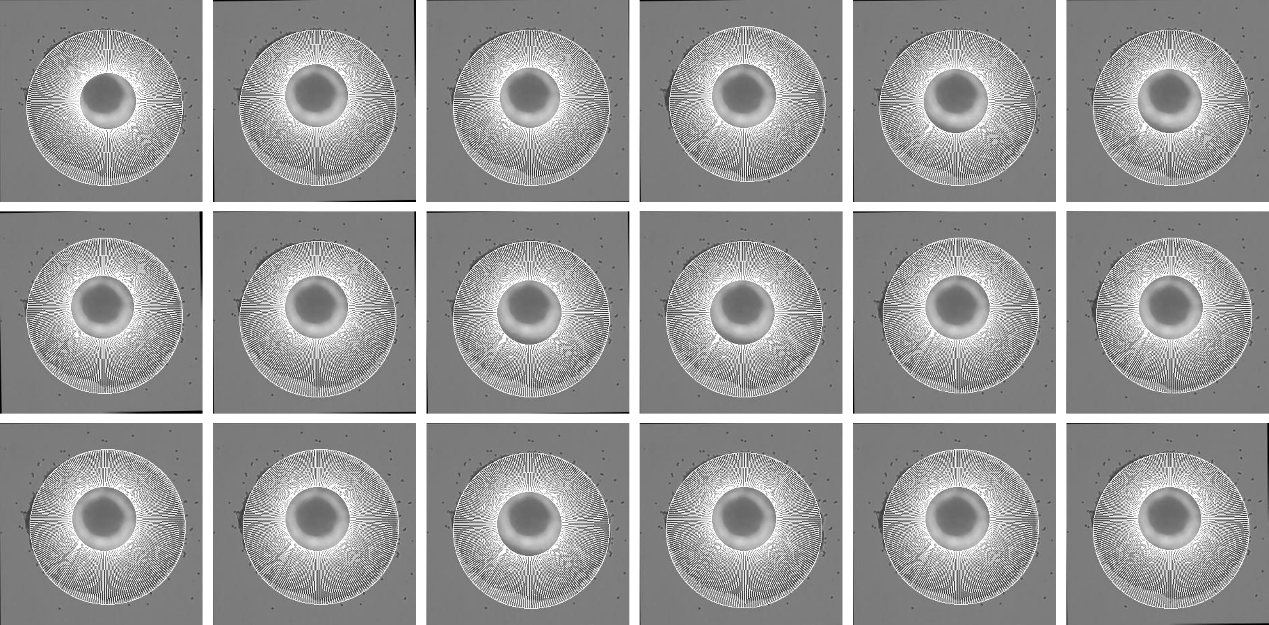


Figure S20 The images with identified outer and inner contours are segmented


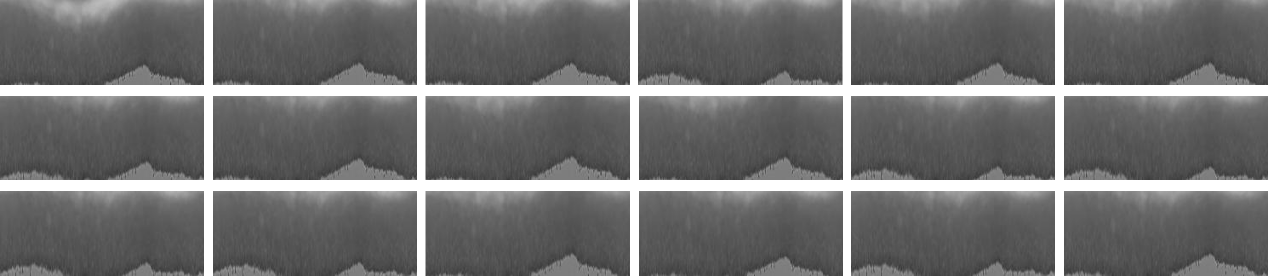


Figure S21 The segmented images are unfolded and encoded


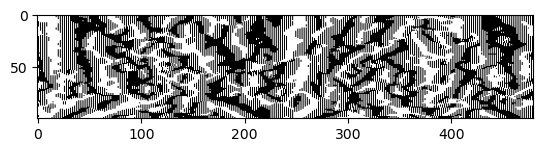


Figure S22 The gray-scale matrix input into the neural network

The shape of the biometric template is 480×100, and the template is flattened into a vector of length 48,000 as the input of the neural network


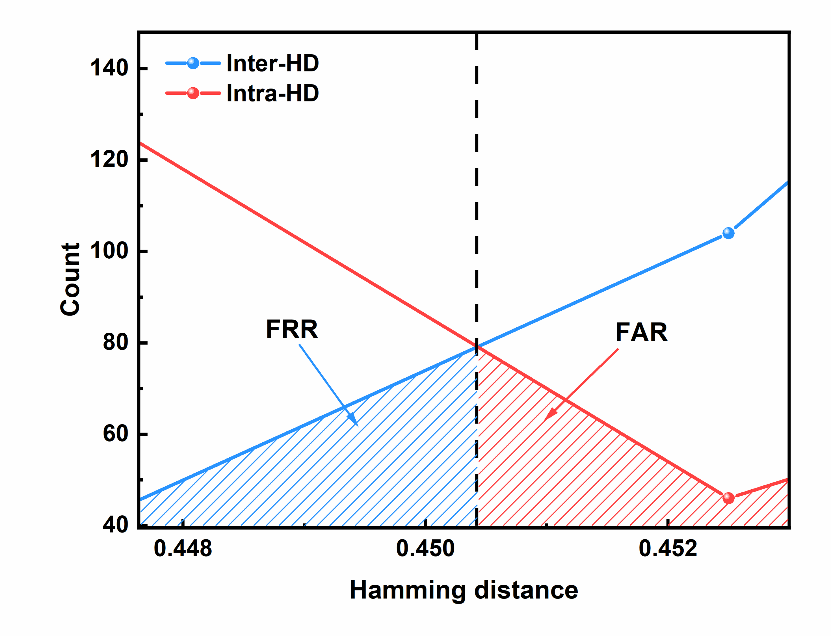


Figure S23 Distribution plots of Inter-HD and Intra-HD


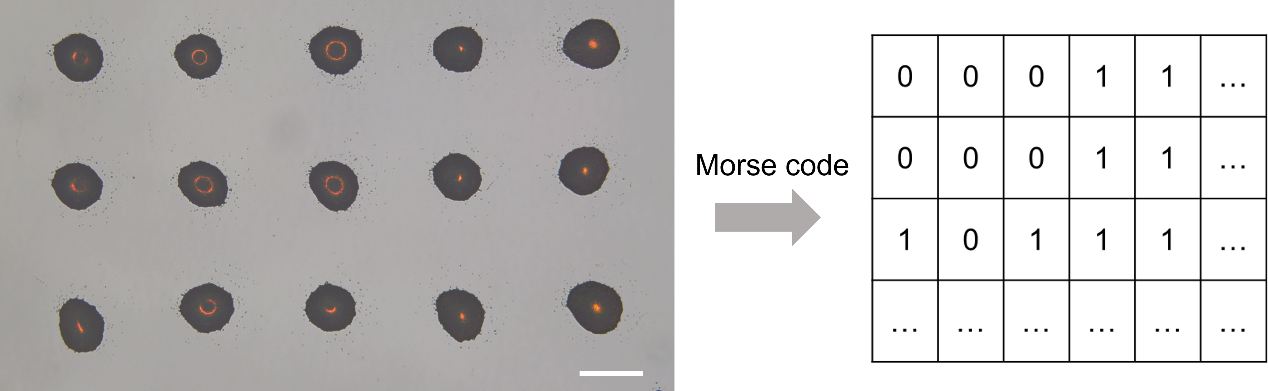


Figure S24 Schematic Diagram of the Morse Encoding Process, with a scale bar of 50 μm.


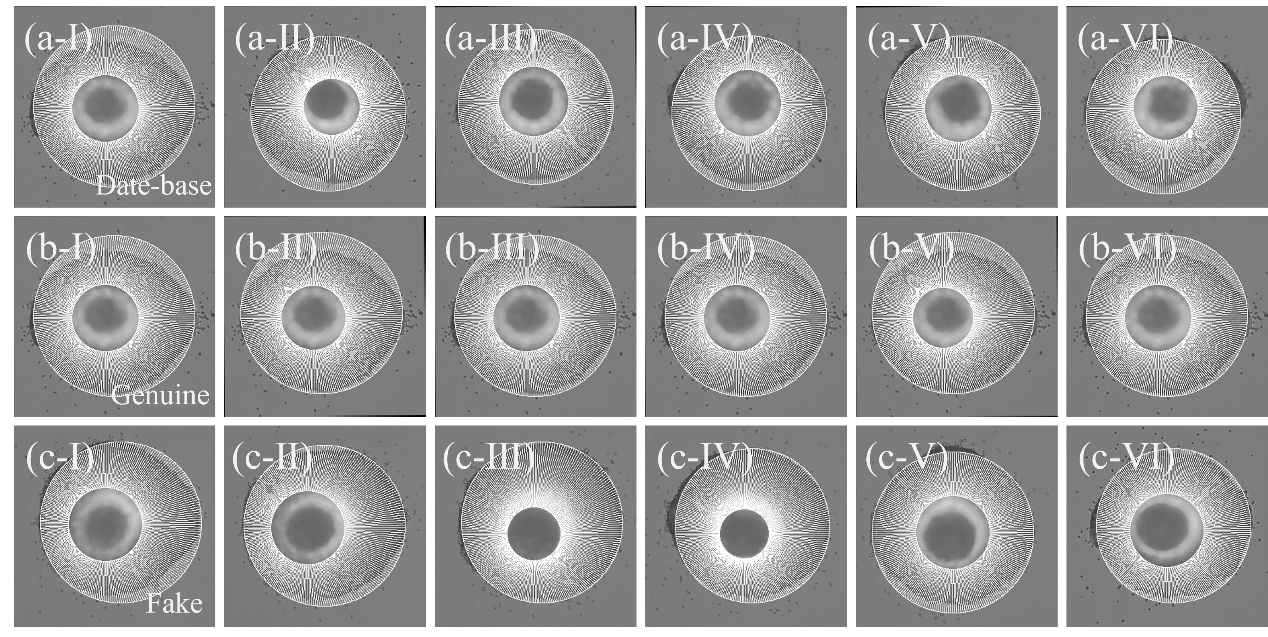


Figure S25 Establishment of the Optical PUF Anti-Counterfeiting System Database

(a-I)-(a-VI) Images in the database, sourced from the manufacturer (genuine products); (b-I)-(b-VI) Images derived from (a-I) in the database, simulating those uploaded by consumers to the cloud; (c-I)-(c-VI) Images fabricated by counterfeiters, not originating from the database (counterfeit products).

Supplementary Text

**Text 1：Supplement to flow field simulation**

Firstly, we assumed that drop evaporation is a quasi-steady state process, and both the liquid and gas flows can be considered as incompressible laminar flow of a Newtonian fluid. Under these assumptions, the governing equations for the evaporating drop and the surrounding gas can be obtained as follows:

The continuity equation for the fluid region is given as follows:

|  | $\nabla\cdot\vec{u}=0$, | (S1$)$ |
| --- | --- | --- |

where $\vec{u}$ is the velocity field of the fluid.

The momentum equation for the fluid region is described using the following:

|  | $\frac{\partial\left( \rho_{l}\vec{V}_{l} \right)}{\partial t}+\vec{V}_{l}\nabla\left( \rho_{l}\vec{V}_{l} \right)=-\nabla p_{l}+\rho_{l}g+\mu_{l}\nabla^{2}\vec{V}_{l},$ | (S2$)$ |
| --- | --- | --- |

where $p_{l}$ is the fluid pressure, $V_{l}$ is the fluid dynamic viscosity, $\rho_{l}$ is the fluid density, and $g$ is the gravitational constant. We utilize the Boussinesq approximation $\rho_{l}=\rho_{0}\left[ 1+\beta\left( T_{0}-T \right) \right]$, where $\rho_{0}$ is the fluid density at temperature $T_{0}$ state，and $\beta$ represents the thermal expansion coefficient.

The energy equation for the fluid region is expressed as follows:

|  | $\rho_{l}C_{l}\frac{\partial T_{L}}{\partial t}+\rho_{l}C_{l}\vec{V}_{l}\cdot\nabla T_{l}=\nabla\left( k_{l}\nabla T_{l} \right),$ | (S3$)$ |
| --- | --- | --- |

where $T_{l}$ represents the liquid temperature, $C_{l}$ is the specific heat capacity of the fluid, $k_{l}$ is the thermal conductivity of the fluid.

The boundary conditions are defined as follows:

When *z* = 0,

|  | $k_{s}\frac{\partial T}{\partial z}=k_{l}\frac{\partial T}{\partial z} \left( 0\leq r\leq R \right),$ | (S4$)$ |
| --- | --- | --- |

where *R* denotes the radius of the droplet.

On the symmetrical axis, *x* = 0,

|  | $\begin{matrix} \frac{\partial T_{1}}{\partial r} & =0 \text{(z>0)} \\ & \\ \frac{\partial T_{s}}{\partial r} & =0 \text{(z<0)} \end{matrix},$ | (S5$)$ |
| --- | --- | --- |

The thermal boundary condition at the free surface can be expressed as follows:

|  | $k_{l}\frac{\partial T_{l}}{\partial\vec{n}}=J\left( r \right)\Delta H,$ | (S6$)$ |
| --- | --- | --- |

where $\Delta H$ represents the latent heat of the droplet. $J\left( r \right)=-D\left( \nabla\cdot\vec{n} \right)C_{\nu}+\left( \vec{V_{g}}\cdot\vec{n} \right)C_{\nu}$ is the evaporation flux, where $D$ indicates the diffusion coefficient, $\vec{V_{g}}$ denotes the velocity in the gas field, $\vec{n}$ is the normal direction, and $C_{\nu}$ is the molar concentration of the gas, which is substituted here with the corresponding saturation vapor pressure ($C_{\nu}=\frac{P_{\mathrm{sat}}\left( T \right)}{RT}$).

On the gas–liquid interface, the shear stress can be expressed as follows:

|  | $\nabla\tau_{l}=\nabla\sigma,$ | (S7$)$ |
| --- | --- | --- |

where $\nabla\tau_{l}$ denotes the shear stress on the gas–liquid interface, and $\nabla\sigma$ represents the surface tension gradient on the interface.

Assuming the surface tension gradient of water decreases linearly with temperature:

|  | $\sigma_{T}\left( T \right)=\sigma_{0}-\left\vert\frac{d\sigma}{dT} \right\vert\left( T-T_{a} \right),$ | (S8$)$ |
| --- | --- | --- |

where $\sigma_{0}$ is the surface tension at temperature $T_{a}$, thus $\nabla\sigma=-\left| \frac{d\sigma}{dT} \right|\nabla T$. When Marangoni flow is neglected, $\nabla\tau_{l}=\nabla\sigma=0$, which implies that $\sigma_{\mathrm{surface}}\approx\sigma_{0}$.

Three different simulation models:

(1) In the “Sole Natural convection” model, Marangoni flow term: *σ*_T_ = 0; Natural convection term: g = 9.8 m/s^2^, interface viscosity = 0.001 Pa.s.m.

(2) In the “Sole Marangoni flow” model, the Marangoni convection term: σ_T_ = 1 × 10^−6^ N/(m·K); Natural convection term: g = 0, interface viscosity = 0.001 Pa.s.m.

(3) In the “Natural convection + Marangoni flow” model, Natural convection term: *g* = 9.8 m/s^2^; Marangoni convection term: *σ*_T_ = 1 × 10^−6^ N/(m·K), interface viscosity = 0.001 Pa.s.m.

**Text 2: PUF Image Processing Procedure**

The optical micrographs of PUF patterns are uniformly cropped to the same dimensions, and their grayscale images are extracted. The cropped grayscale images are then used as the original image features and serve as inputs. After undergoing the following series of operations, the final image features for the neural network are obtained.

**1. Canny Edge Detection**

The goal of Canny edge detection is to identify an optimal edge detection method that achieves low error rates, accurate edge localization, and minimal response. PUF patterns are initially processed using Canny edge detection. The Canny edge detection algorithm first applies Gaussian filtering to remove noise from the grayscale image and smooth the image, thereby avoiding the detection of false edges. Subsequently, gradient operators such as the Sobel operator are used to calculate the gradient magnitude and direction of the image, representing edge strength and edge direction, respectively. True edges are then refined and extracted through non-maximum suppression and threshold detection. The threshold value is set to 0.2.

After Canny edge detection, each input PUF grayscale image yields a binary matrix, where 1 or 0 indicates whether a pixel is part of an edge or not. Through Canny edge detection, the inner and outer contour images in Figure S18 and Figure S19 are obtained.

**2. Hough Transform**

The Hough transform is an algorithm used to detect geometric shapes in binary images. The input for the Hough transform is the binary matrix obtained from Canny edge detection. Since the PUF patterns in this work mimic the iris, the goal of the Hough transform is to extract circles corresponding to the iris shape. The radius for circle detection in the Hough transform is set to 45, and the threshold is set to 4.

Through the Hough transform, the circles at two positions of the iris in Figure S20 are obtained.

**3. Localization Marking**

Localization is a core aspect of iris recognition, aiming to locate the iris, pupil, and the noise region between them. By localizing these regions and marking their positions in the image as white (pixel value of 255), the positions are identified, resulting in Figure S21.

**4. Normalization and Feature Extraction**

The marked regions are transformed into polar coordinates to normalize the image, reducing interference from varying imaging conditions and facilitating subsequent feature extraction. After polar coordinate transformation and normalization, a Gabor filter is used to extract texture information from the iris image. Following filtering, the filter's output is binarized to generate a biometric template. This template consists of the texture features of the iris image, primarily the real and imaginary components. Additionally, a mask is applied to record regions with low-amplitude noise based on the filter's amplitude information.

Through the above steps, a biometric template for each iris image is established. This template records the texture features of the iris region, thereby preparing the data for subsequent neural network learning and matching (Figure S22).

**Text 3: PUF Training Process**

Following the aforementioned operations, a representative feature sample, which characterizes the original pattern, is extracted from all PUF patterns and serves as the input to the neural network. All samples are randomly shuffled, with 80% allocated for training and 20% for validation.

Subsequently, a Back Propagation Neural Network (BPNN) is constructed to differentiate between genuine and counterfeit patterns. The neural network comprises an input layer, a single hidden layer, and an output layer, with the hidden layer containing 200 neurons.

During the training process, the number of epochs is set to 100, the learning rate is set to 0.01, and the Levenberg-Marquardt algorithm is employed as the optimization method.

**Text 4：Randomness Verification Parameters**

**1. Bit Uniformity**

Bit uniformity is an indicator used to measure whether the distribution of bits in a bit sequence is uniform. It reflects whether the frequencies of 0s and 1s in a given bit sequence are approximately equal. A value closer to 0.5 indicates better randomness in the data. For fields such as information security, data compression, and encryption, uniformity is a critical metric for assessing randomness. It can be expressed using the following formula:

|  | $\text{Bit uniformity}=\frac{1}{s}\sum_{l=1}^{s} K_{l}$ | (S9) |
| --- | --- | --- |

where $K_{l}$ represents the response (0 or 1) at the $l$ position of an array with $S$ positions

**2. Information entropy**

Information entropy serves as a crucial measure of the uncertainty inherent in information or the average information content. The closer its value approaches 1, the better the randomness of the data. It can be expressed as follows:

|  | $\text{Information entropy}=-\sum_{i=1}^{n} P(x_{i})\log_{b}P(x_{i})$ | (S10) |
| --- | --- | --- |

where $P(x_{i})$ denotes the probability of event $x_{i}$ occurring.

**3. Correlation coefficient**

The correlation coefficient represents the strength and direction of the linear relationship between two variables. A value of 1 indicates a perfect positive correlation, -1 indicates a perfect negative correlation, and 0 indicates no correlation. It can be expressed using the following formula:

|  | $\text{Correlation coefficient}=\frac{\sum(X-\bar{X})(Y-\bar{Y})}{\sqrt{\sum(X-\bar{X})^{2}\sqrt{(Y-\bar{Y})^{2}}}}$ | (S11) |
| --- | --- | --- |

where $X$ and $Y$ represent two distinct sets of binary variables, while $\bar{X}$ and $\bar{Y}$ denote the averages.

**4. NIST Test**

The NIST test (National Institute of Standards and Technology) is a series of statistical tests developed by the National Institute of Standards and Technology, comprising a total of 15 test items. Due to sample size limitations, the first nine tests are conducted, which include the frequency test, frequency block test, runs test, longest run test, discrete Fourier transform test, serial test, Markov test, matrix rank test, and autocorrelation test. Each test item generates a P-value. If the P-value exceeds 0.01, the sequence is considered to meet the randomness requirements for that test.

**5. Hamming distance**

The Hamming distance quantifies the discrepancy between two distinct sets by measuring the number of corresponding elements that differ at each position in two strings of equal length. It can be expressed using the following formula:

|  | $\text{Hamming distance}=\frac{\text{\# of different bits }}{\text{\# of total bits}}$ | (S12) |
| --- | --- | --- |

where $\#$ denotes a string.

**Text 5: Machine learning algorithm implements details**

First extract the grayscale image of the PUF pattern, and then use the Canny edge detection algorithm and Hough transform to find the location of the pupil and iris. Then we flatten the image of the iris area into a two-dimensional matrix and normalize it. Finally, we use Gabor convolution to extract the features of this two-dimensional matrix as the biometric template of each PUF image. The shape of the biometric template is 480×100, and the template is flattened into a vector of length 48,000 as the input of the neural network. All the PUF samples are divided into two parts labeled genuine or fake. And then these samples are randomly shuffled and divided into training set and validation set.

After the feature engineering above, we established a three-layers back propagation neural network. The first layer contains 48,000 neurons, and the second layer contains 200 neurons, the last output layer contains only 1 neuron.

The learning rate is set to 0.01, learning epoch is set to 100, early stopping is employed.
